# HDAC6 selective inhibition of melanoma patient T-cells augments anti-tumor characteristics

**DOI:** 10.1186/s40425-019-0517-0

**Published:** 2019-02-06

**Authors:** Andressa S. Laino, B. C. Betts, A. Veerapathran, I. Dolgalev, A. Sarnaik, S. N. Quayle, S. S. Jones, J. S. Weber, David M. Woods

**Affiliations:** 1 NYU Langone Health, 522 First Avenue, 1306 Smilow Research Building, New York, NY 10016 USA; 2 H. Lee Moffitt Cancer Center & Research Institute, Tampa, FL USA; 3 Acetylon Pharmaceuticals, Boston, MA USA; 4 Regenacy Pharmaceuticals, Boston, MA USA

**Keywords:** HDAC inhibitor, HDAC6, ACY-1215, ACY-241, Melanoma, T-cells, Central memory, Exhaustion, Regulatory T-cell

## Abstract

**Background:**

Therapies targeting anti-tumor T-cell responses have proven successful in the treatment of a variety of malignancies. However, as most patients still fail to respond, approaches to augment immunotherapeutic efficacy are needed. Here, we investigated the ability of histone deacetylase 6 (HDAC6)-selective inhibitors to decrease immunosuppression and enhance immune function of melanoma patient T-cells in ex vivo cultures.

**Methods:**

T-cells were harvested from peripheral blood or tumor biopsies of metastatic melanoma patients and cultured in the presence of pan-, class-specific or class-selective histone deacetylase (HDAC) inhibitors. Changes in cytokine production were evaluated by Luminex and intracellular flow cytometry staining. Expression of surface markers, transcription factors, protein phosphorylation, and cell viability were assessed by flow cytometry. Changes in chromatin structure were determined by ATAC-seq.

**Results:**

T-cell viability was impaired with low doses of pan-HDAC inhibitors but not with specific or selective HDAC inhibitors. The HDAC6-selective inhibitors ACY-1215 (ricolinostat) and ACY-241 (citarinostat) decreased Th2 cytokine production (i.e. IL-4, IL-5, IL-6, IL-10 and IL-13). Expansion of peripheral blood T-cells from melanoma patients in the presence of these inhibitors resulted in downregulation of the Th2 transcription factor GATA3, upregulation of the Th1 transcription factor T-BET, accumulation of central memory phenotype T-cells (CD45RA-CD45RO + CD62L + CCR7+), reduced exhaustion-associated phenotypes (i.e. TIM3 + LAG3 + PD1+ and EOMES+PD1+), and enhanced killing in mixed lymphocyte reactions. The frequency, FOXP3 expression, and suppressive function of T regulatory cells (Tregs) were decreased after exposure to ACY-1215 or ACY-241. Higher frequencies of T-cells expressing CD107a + IFNγ+ and central memory markers were observed in melanoma tumor-infiltrating lymphocytes (TIL), which persisted after drug removal and further expansion. After ACY-1215 treatment, increased chromatin accessibility was observed in regions associated with T-cell effector function and memory phenotypes, while condensed chromatin was found in regions encoding the mTOR downstream molecules AKT, SGK1 and S6K. Decreased phosphorylation of these proteins was observed in ACY-1215 and ACY-241-treated T-cells. AKT- and SGK1-specific inhibition recapitulated the increase in central memory frequency and decrease in IL-4 production, respectively, similar to the observed effects of HDAC6-selective inhibition.

**Conclusions:**

HDAC6-selective inhibitors augmented melanoma patient T-cell immune properties, providing a rationale for translational investigation assessing their potential clinical efficacy.

**Electronic supplementary material:**

The online version of this article (10.1186/s40425-019-0517-0) contains supplementary material, which is available to authorized users.

## Introduction

Populations of cancer-reactive T-cells can be found in the tumor microenvironment of many patients, but their ability to eliminate tumor is limited by diverse immunosuppressive mechanisms. Th2 polarization of T-cells impairs cytolytic effector responses [1], which is associated with decreased therapeutic efficacy [2], and reduced survival [3]. Regulatory T-cells (Tregs) hinder anti-tumor immune responses through mechanisms including production of immunosuppressive cytokines (e.g. TGFβ), downregulation of co-stimulation by antigen-presenting cells, and killing of effector T-cells [4]. Additionally, tumor-reactive T-cells are often dysfunctional [5], having increased expression of inhibitory checkpoint molecules [6] and altered transcriptional profiles [7]. Overcoming these immunosuppressive influences remains an important prerequisite for increasing the efficacy of cancer immunotherapies.

Histone deacetylases (HDACs) are a family of epigenetic regulatory enzymes distinguished by their ability to remove acetyl groups from the lysine residues of histone tails, thereby condensing chromatin structure and suppressing gene expression. Recent evidence has demonstrated that HDACs are also able to deacetylate other substrates beside histones [8]. The zinc-dependent, or classical HDACs are divided into four classes: class I (HDAC1, HDAC2, HDAC3, HDAC8), class IIA (HDAC4, HDAC5, HDAC7, HDAC9), class IIB (HDAC6 and HDAC10) and class IV (HDAC11).

Several pan-HDAC inhibitors (e.g. LBH589, PDX101) are FDA-approved for the treatment of hematologic malignancies. Class-selective and HDAC-specific inhibitors have also been developed, and several are being evaluated in clinical trials. HDAC inhibitors (HDACi) can promote direct tumor cytotoxicity and studies have demonstrated that they alter tumor immunogenicity [9, 10]. Research addressing the direct effects of HDACi on T-cells has shown disparate effects, dependent on the timing, potency, dose and specificity of the inhibitor used [11].

To better understand the impact of HDACi on human T-cells, we analyzed the effects of selective HDAC6 inhibition ex vivo on the immune properties of T-cells derived from melanoma patient peripheral blood mononuclear cells (PBMC) and tumor biopsies. We found that treatment with the HDAC6-selective inhibitors ACY-1215 (ricolinostat) and ACY-241 (citarinostat) reduced T-cell immunosuppressive functions (i.e. Treg suppression, Th2 cytokine production and exhaustion-associated phenotypes) while enhancing effector function (i.e. increased T-BET expression, accumulation of T-cells expressing a central memory phenotype, increased IFNγ and CD107a expression, and cytoxicity). Collectively, these results demonstrate novel effects of HDAC6-selective inhibition on the phenotype and function of melanoma patient T-cells.

## Materials and methods

### Human samples

PBMC were obtained by leukapheresis from metastatic melanoma patients prior to treatment. TILs were obtained from surgical biopsies of melanoma metastases by culturing tumor fragments in 6000IU/mL IL-2 for 2–3 weeks [12]. All protocols were approved by the Institutional Review Board at H. Lee Moffitt Cancer Center (IRB 106509, 107,273; Tampa, FL). Cells were obtained under a Materials Transfer Agreement. Samples were coded with anonymized 5-digit numbers and their identity was unknown to those performing the experiments. PBMCs were separated using 1.077g/mL Ficoll Histopaque (ThermoFisher Scientific, Waltham, MA), according to the manufacturer. CD3+ T-cells were isolated through EasySep magnetic negative isolation (StemCell; Vancouver, BC, Canada) and cultured with 10% AB human serum (Omega Scientific; Tarzana, CA), beta-mercaptoethanol, non-essential amino acids, HEPES, penicillin, streptomycin and gentamicin supplemented X-VIVO media (Corning; Corning, NY).

### Inhibitors

ACY-1215, LBH589, Tubastatin A, MS275, MGCD0103, GSK650394, A-674563, and Rapamycin were purchased from Selleck Chemicals (Houston, TX). ACY-241 was provided by Acetylon Pharmaceuticals (Boston, MA). Inhibitors were reconstituted in dimethyl sulfoxide (DMSO) and stored at -80°C. Thaw and dilution were performed immediately prior to usage.

### T-cell cytokine production

T-cells were treated for one hour with inhibitors, activated with αCD3/CD28 Dynabeads (ThermoFisher) at 1:1 ratio and cultured for 72 h. Supernatant was collected for cytokine production assessment by Luminex assay (R&D Systems; Minneapolis, MN), according to the manufacturer. A Luminex 200 instrument (R&D) was used for sample acquisition.

### Flow cytometry analyses

Surface staining was performed in FACS buffer (PBS, 2mM EDTA, 2% fetal bovine serum), for 30 min at 4°C. Antibodies used are listed in Additional file 1: Table SI. DAPI was used to determine viability. For intracellular staining, the Foxp3 transcription factor staining buffer set (eBioscience) was used. Live Dead dyes (ThermoFisher) were used to assess viable cells. Acquisition of cells was performed on an LSR II (BD) or Attune NxT (ThermoFisher) instruments. FlowJo v10 software was used for data analyses.

### Treg expansion and suppression assays

Melanoma patient PBMC-derived natural Tregs (nTregs) were isolated using the CD4 + CD127lowCD25+ EasySep isolation kit (StemCell). nTregs were treated with ACY-1215 500nM or DMSO and cultured for five days in 10μg/mL OKT3-coated plates, 2μg/mL anti-CD28 (Biolegend), 10ng/mL TGFβ (R&D), 300IU/mL IL-2 (Aldesleukin; Prometheus Laboratory, San Diego, CA). nTregs were washed and co-cultured with either CD4 + CD25- T-conventional (Tcon) or CD8+ Tcons at 1:5 ratio (Treg:Tcon), plus αCD3/CD28 Dynabeads. Tcon proliferation was assessed after five days co-culture by flow cytometry assessment of CD8 + Ki67+ or CD4 + FOXP3-Ki67+, as indicated.

Inducible Tregs (iTregs) were generated by isolating CD4+ naïve T-cells from melanoma patient PBMCs via EasySep purification, and expansion for five days in 10μg/mL OKT3-coated plates, 2μg/mL anti-CD28, 300IU/mL IL-2, 10ng/mL TGFβ, and 100nM Rapamycin. Suppression of Tcon proliferation was performed by co-culturing autologous CD8+ Tcons with iTregs at the indicated ratios, plus αCD3/CD28 Dynabeads. Flow cytometry for CD8 + Ki67+ was performed to determine Tcon proliferation.

### TIL expansion

Tumor fragments were cultured in media containing 6000IU/mL IL-2 [12]. Expanded TILs were frozen under liquid nitrogen. Thawed TILs were cultured for one week with 6000IU/mL IL-2 and DMSO or HDAC inhibitors, at the indicated doses. For experiments involving expansion of activated TILs, HDAC inhibitors and DMSO were washed out after one week, and cells were activated with αCD3/CD28 Dynabeads (ThermoFisher) for additional seven days.

### Mixed lymphocyte reaction

Melanoma patient CD3+ T-cells were expanded in 6000IU/mL IL-2 for one week with DMSO, ACY-1215 500nM or ACY-241500nM, then washed and co-cultured with CFSE-stained (0.1uM), irradiated (1000 rads) allogeneic PBMC cells, at the indicated ratios. After 96 h, viable target cell counts were determined by flow cytometry.

### Assay for transposase-accessible chromatin with high-throughput sequencing (ATAC-seq)

CD3+ T-cells isolated from melanoma patient PBMCs were expanded in 6000IU/mL IL-2 for one week with DMSO or ACY-1215 500nM, then activated via αCD3/CD28 for 24 h. 50,000 cells per group were used to generate ATAC-seq libraries, as previously described [13]. Briefly, fastq files were generated through ATAC-seq. Capture and sequencing were aligned to the mouse genome build hg19 using BWA. Polymerase chain reaction duplicated reads were filtered out using Picard software tool (MarkDuplicates.jar). Significant peaks were then identified through Zero Inflated Negative Binomial Algorithm (ZINBA), a computational method designed to call regions of the genome enriched for sequencing reads originating from a diverse array of biological experiments. The resulting peak intensity information was combined with peak density data using the program DiffBind that identified differential open chromatin sites between experimental and control data. These differential binding regions with open and closed chromatin were then annotated using ANNOVAR.

### Statistical analyses

GraphPad Prism 7 software was used for statistical analyses, and *p*-values ≤0.05 were considered significant. Paired, two-tailed, t-tests were used to determine statistical significance between control versus treatment groups, unless otherwise indicated. ATAC-seq *p*-values were false discovery rate (FDR)-adjusted and values ≤0.05 considered significant.

## Results

### Selective inhibition of HDAC6 did not impair T-cell viability

**Fig. 1:**
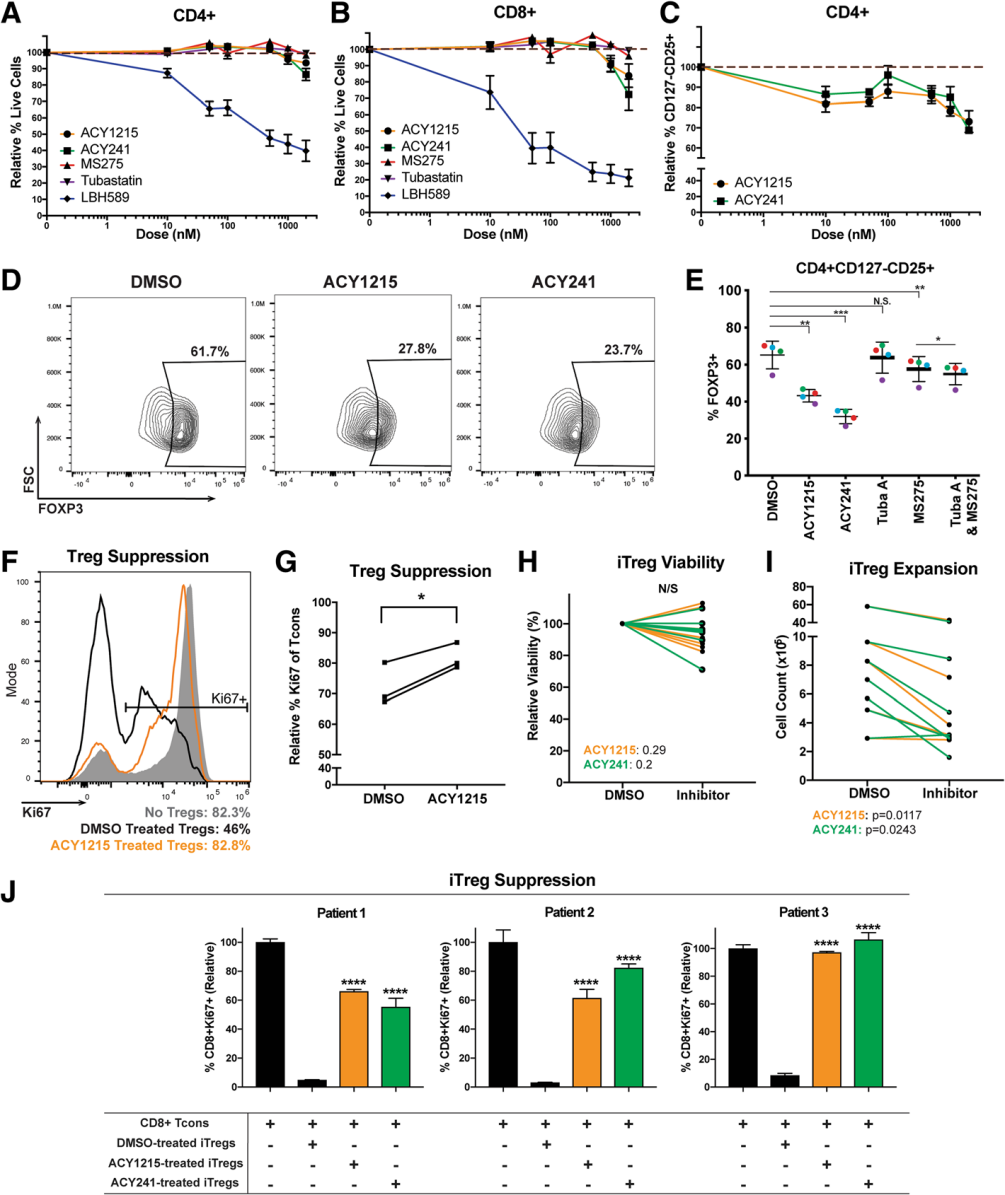
ACY-1215 and ACY-241 reduce Treg percentages and suppressive function. (**a**) CD4+ and (**b**) CD8+ melanoma patient T-cells were cultured for 72 h with 100IU/mL IL-2, plus ACY-1215 (circles), ACY-241 (squares), MS275 (triangles), Tubastatin A (inverted triangles), LBH589 (diamonds), or DMSO. Viability was assessed by DAPI. Representative experiment assessing three samples is shown; six patients were assessed over two experiments. (**c**) CD4 + CD127^lo^CD25+ viability was assessed in T-cells cultured with 100IU/mL IL-2 for 72 h, plus ACY-1215 (circles) or ACY-241 (squares). Representative experiment assessing three patient samples is shown; eight patients were assessed over three experiments. (**a**-**c**) Y-axis displays the mean viability (±SEM) relative to DMSO; X-axis indicates inhibitor doses. (**d**) FOXP3 expression was assessed in CD4 + CD127^lo^CD25+ Tregs by flow cytometry for ACY-1215 or ACY-241 treatments (500nM). Representative contour plots for one sample are shown; four patients were assessed. (**e**) FOXP3 expression was evaluated in additional four samples over two experiments, each denoted by a color, treated with the indicated nhibitors. (**f**) DMSO (black histogram) or ACY-1215 500nM (orange histogram) pre-treated nTregs from one patient were co-cultured with autologous CD8+ Tcons (1:5 Treg:CD8+). Ki67 expression in CD8+ T-cells was assessed after five days. Control (only CD8+, no nTregs) is shown in grey. (**g**) nTreg suppression was assessed in three samples in an independent experiment, using CD4+ Tcons. The percentage of Ki67 expressing CD4 + FOXP3- Tcon cells is shown. (**h-i**) CD4+ naïve-derived iTregs from seven patient samples were expanded and treated with DMSO or 500nM ACY-1215 over four experiments. (**h**) Relative viability and (**i**) cell counts were assessed by Trypan Blue seven days after culture. (**j**) iTregs from three patients were expanded and treated with DMSO or 500nM ACY-1215 over three experiments. Autologous CD8+ T-cells were co-cultured with iTregs, activated for 48-72h, and assessed for Ki67 expression. Proliferation percentages relative to control (Tcons only) are shown. *p < 0.05, **p < 0.01, ***p < 0.001, ****p < 0.0001

Studies have shown that suppression of HDAC activity using small molecule inhibitors may decrease T-cell survival [14]. We evaluated several HDAC inhibitors with differing specificities for their impact on T-cell viability in vitro. At low concentrations (i.e. 10nM), pan-HDAC inhibition with LBH589 resulted in increased cell death (Fig. 1A-B, blue lines), which was also seen with other pan-HDACi (SAHA and quisinostat) (data not shown). In contrast, viability was not impaired at concentrations up to 1uM with the class I HDACi MS275 (red lines), the HDAC6-specific inhibitor Tubastatin A (purple lines), or the HDAC6 selective inhibitors ACY-1215 (orange lines) and ACY-241 (green lines). ACY-1215 and ACY-241 are structural analogues with a similar inhibitory profile [15].

To verify the class specificity of the inhibitors evaluated, T-cells were cultured with DMSO (control), class I HDACi (MGCD0103), HDAC6-selective HDACi (ACY-1215) or HDAC6-specific HDACi (Tubastatin A). Cells were then assessed for acetylation of α-tubulin, a marker of HDAC6 inhibition, and acetylated histone 3, a marker of inhibition of HDAC1, HDAC2 and HDAC3, members of class I HDACs (Additional file 2: Figure S1). Treatment with the class I-specific inhibitor MGCD0103 showed no difference in levels of acetylated α-tubulin relative to DMSO but resulted in increased levels of acetylated histone 3. Tubastatin A showed a dose-dependent increase in the levels of acetylated α-tubulin but had minimal impact on histone 3 acetylation. ACY-1215 treatment resulted in increased acetylated α-tubulin like those seen at equivalent doses of Tubastatin A, and increased histone 3 acetylation levels, suggesting that ACY-1215 was a selective, rather than a specific HDAC6 inhibitor. This was consistent with previous results showing inhibition of class I HDACs by ACY-1215 [16].

### HDAC6 selective inhibition reduced Treg yield, FOXP3 expression and suppressive function

Pan-HDAC inhibition can augment Treg generation and suppressive function [17], which is attributed to inhibition of class II HDACs [18, 19], including HDAC6 inhibition [20]. In contrast, class I HDAC [21] and selective HDAC6 inhibition [22, 23] have been shown to decrease Treg expansion. The differential impact of class I and HDAC6 inhibition on Tregs led us to investigate the effects of ACY-1215 on Treg suppression. We evaluated Tregs using the surface marker set CD4 + CD127^-/low^CD25+ [24] in T-cells harvested from melanoma patient PBMC and treated with ACY-1215 or ACY-241. CD127^-/low^CD25+ percentages in CD4+ T-cells were reduced after treatment with ACY-1215 relative to DMSO, at concentrations ranging from 10nM to 2uM (Fig. 1 C). The flow cytometry gating strategy for Tregs is shown in Additional file 2: Figure S2A. We next evaluated the effects of HDAC6-selective inhibition on expression of the Treg master transcription factor FOXP3 [25] in CD4 + CD127^-/low^CD25+ Tregs. We found that the percentages of FOXP3-expressing Tregs were reduced in ACY-1215-treated (*p* = 0.004) and ACY-241-treated (*p* = 0.001) cultures relative to DMSO (Fig. 1 D-E). In comparison, MS275 reduced FOXP3 expression to a lesser degree (*p* = 0.003) while Tubastatin A did not have an effect. We also evaluated if the combination of a class I inhibitor, MS275, and HDAC6i, Tubastatin A, could recapitulate the effects seen with ACY-1215 and ACY-241. While the combination significantly decreased FOXP3 expression relative to MS275 alone (*p* = 0.014), the magnitude of downregulation was minimal (Fig. 1E).

To determine whether the decrease in FOXP3 induced by ACY-1215 and ACY-241 resulted from transcriptional downregulation, CD4+ T-cells were cultured with or without αCD3/CD28 activation and treated with ACY-1215 or ACY-241. *foxp3* message was downregulated in both non-activated and activated samples (Additional file 2: Figure S2B-C).

Given the observed reduction in FOXP3 protein and message induced by ACY-1215 and ACY-241, we evaluated alterations in histone acetylation of transcription factor binding regions of the *foxp3* gene. Increased levels of acetylated histone 3 were found at known RUNX3, SMAD3 and GATA3 binding regions of the *foxp3* gene in ACY-1215-treated cells relative to DMSO (Additional file 2: Figure S2D).

To determine the impact of HDAC6-selective inhibition on nTreg suppressive function, isolated nTregs (CD4 + CD127^-/low^CD25+) were expanded with ACY-1215, washed, co-cultured with autologous CD8+ T-cells (Tcons) and activated via αCD3/CD28. Figure 1F shows that ACY-1215-treated nTregs had higher levels of Ki67 expression in CD8+ Tcons (i.e. lower nTreg suppression) compared to DMSO-treated nTregs. Tcon proliferation was likewise evaluated using autologous conventional CD4+ Tcons (CD4 + FOXP3-). ACY-1215-expanded nTregs had reduced suppressive capacity of CD4 + FOXP3- Tcon proliferation compared to control-treated Tregs (*p* = 0.0244) (Fig. 1G).

We evaluated whether inducible Tregs (iTregs) polarized from naïve CD4+ T-cells in the presence of HDAC6-selective inhibitors displayed a less suppressive phenotype. Although no changes were observed in overall viability (Fig. 1H), the yield of iTregs was reduced relative to DMSO controls (Fig. 1I; *p* < 0.05). ACY-1215 and ACY-241 pre-treated iTregs displayed decreased suppressive function (~ 60–100% CD8+ Tcon Ki67+), in contrast to the suppression seen in DMSO control pre-treated iTregs (~ 10% CD8+ Tcon Ki67+; *p* < 0.0001) (Fig. 1J).

We also assessed the effects of HDAC6-selective inhibition on nTregs harvested from healthy donor PBMCs [26]. Equivalent cell numbers (CD4 + CD127^-/low^CD25+; 2.4 × 10^5^ cells, healthy donor 1; 2.5 × 10^5^ cells, healthy donor 2) from donors were expanded in vitro with allogeneic monocyte-dendritic cells, with the addition of ACY-1215. ACY-1215 reduced the total number of expanded allogeneic Tregs in comparison with controls (donor 1: DMSO 1.44 × 10^6^, ACY-1215 6.4 × 10^4^; donor 2: DMSO 1.42 × 10^6^, ACY-1215 1.01 × 10^6^) (Additional file 2 Figure S2E). ACY-1215-treated nTregs displayed reduced suppressive capacity relative to DMSO-treated controls (healthy donor 1: 74% suppression ACY-1215 versus 90% DMSO, *p* = 0.014; healthy donor 2: 85% suppression ACY-1215 versus 95% DMSO, *p* = 0.063) (Additional file 2 Figure S2F).

### HDAC6 selective inhibition impaired Th2 cytokine production

**Fig. 2:**
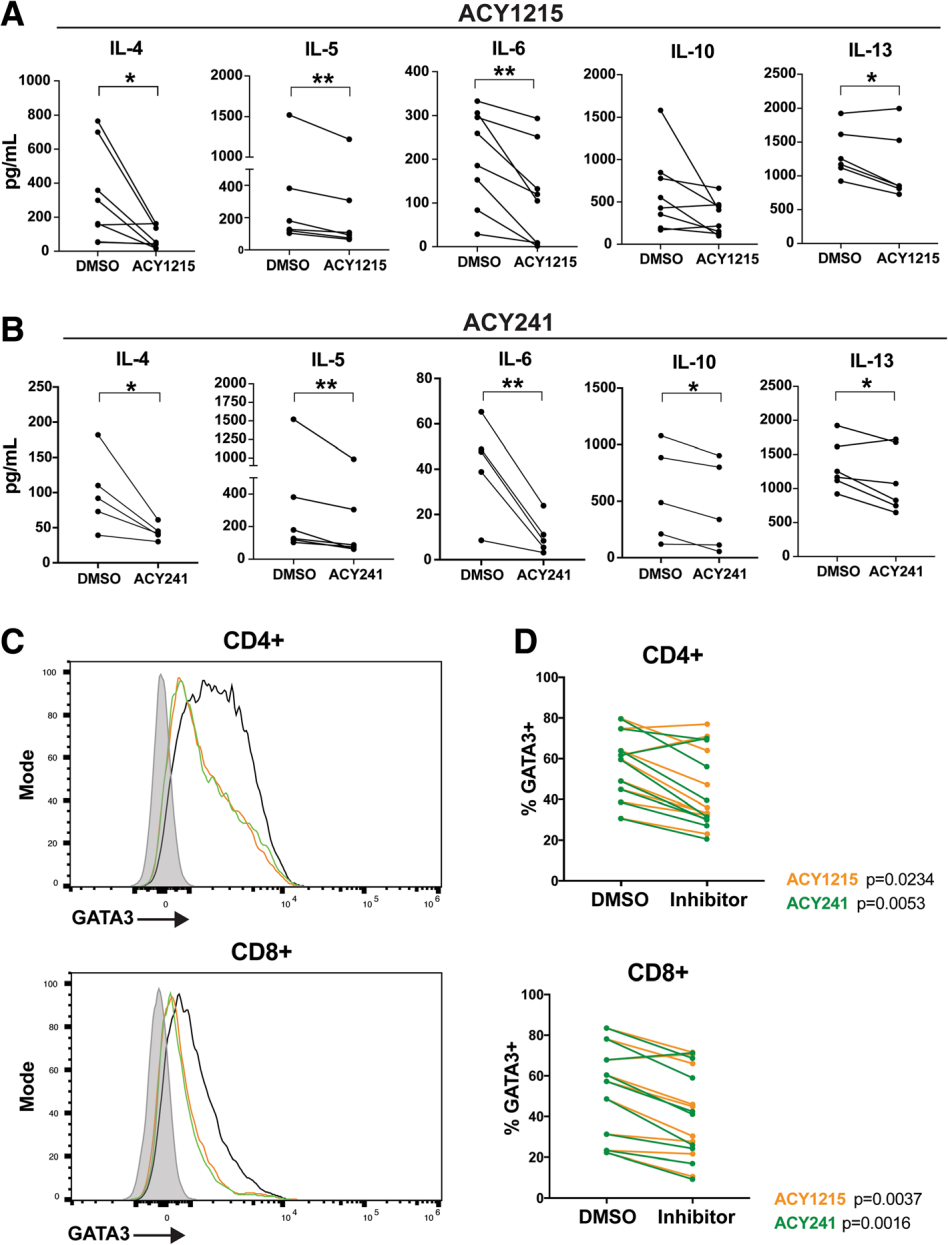
ACY-1215 and ACY-241 decrease Th2 cytokine production by T-cells. T-cells isolated from melanoma patient PBMC samples were treated with DMSO, (**a**) ACY-1215 500nM, or (**b**) ACY-241500nM, and activated for 72 h. Supernatant cytokines were assessed by Luminex. At least five patient samples were evaluated across three experiments for each cytokine and HDACi treatment assessed. Each paired line represents an individual patient sample. *p < 0.05, **p < 0.01. (**c**) GATA3 expression was assessed by flow cytometry in T-cells treated with DMSO, ACY-1215 500nM, or ACY-241500nM, and activated for 72 h. Representative histograms for CD4+ and CD8+ T-cells are shown for DMSO (black), ACY-1215 (orange), ACY-241 (green) and fluorescence minus one (FMO) control (grey). (**d**) CD4+ and CD8+ T-cells expressing GATA3 in DMSO control and paired inhibitor-treated samples are graphed. (**c**-**d**) Results shown are from nine patients evaluated across three experiments

Pan-HDAC inhibitors can impair Th1 cytokine expression while promoting Th2 cytokine production [27]. To assess the impact of HDAC6-selective inhibition on Th1/Th2 T-cell function, T-cells were treated with ACY-1215 or ACY-241, activated via αCD3/CD28, and evaluated for secreted cytokines by Luminex. ACY-1215 and ACY-241 respectively decreased the expression of IL-4 (*p* = 0.0279, *p* = 0.0418), IL-5 (*p* = 0.0061, *p* = 0.0099), IL-6 (*p* = 0.0044, *p* = 0.0091), and IL-13 (*p* = 0.0341, *p* = 0.0421) (Figs. 2A-B). Reduced IL-10 production was observed in ACY-241-treated samples (*p* = 0.0204) and trended towards reduction in ACY-1215-treated T-cells (*p* = 0.08). As a comparison, we measured cytokine secretion after HDAC6-specific and class I HDAC inhibition by tubastatin A and MS275. As shown in Additional file 2 Figsure S3A-B, Tubastatin A reduced IL-5 (*p* = 0.0081) and IL-13 (*p* = 0.0053) production, without altering IL-4, IL-6 and IL-10 levels. MS275 also decreased IL-13 expression (*p* = 0.0171), but increased IL-10 secretion (*p* = 0.0172); no changes in IL-4, IL-5 or IL-6 production were observed. Secreted levels of the Th1 cytokines IFNγ, TNF and IL-2 by total T-cells were not altered by ACY-1215, ACY-241, Tubastatin A, or MS275 at the concentrations assessed (Additional file 2: Figsure S3C-F).

Given the observed reduction in Th2 cytokine production (e.g. IL-4, IL-5, IL-6, IL-13) induced by ACY-1215 and ACY-241, we evaluated their impact on expression of the T-cell polarizing Th2 transcription factor GATA3 [28]. Treatment with ACY-1215 and ACY-241 reduced the levels of GATA3 in activated CD8+ (*p* = 0.0037, *p* = 0.016) and CD4+ (*p* = 0.0234, p = 0.0053), as shown by a representative histogram (Fig. 2C) and paired analysis (Fig. 2D).

### T-cell expansion in HDAC6 selective inhibitors reduced GATA3 and increased T-BET expression

To assess whether the effects of these inhibitors were maintained after prolonged exposure, T-cells were expanded in the presence of ACY-1215 or ACY-241 in a manner similar to that used in clinical protocols of ex vivo T-cell expansion for adoptive T-cell therapy [12]. Briefly, T-cells from melanoma patient PBMCs were cultured for one week in the presence of 6000IU/mL IL-2 plus ACY-1215 or ACY-241. For evaluation of transcription factors, cells were then activated for 24 h. Similar to observations in short-term cultures, a decrease in GATA3 levels was seen after treatment with ACY-1215 or ACY-241 in CD8+ (*p* = 0.03, *p* = 0.033) and CD4+ (*p* = 0.019, *p* = 0.035) T-cells compared to DMSO (Additional file 2 S4A-B).

We next evaluated the impact of HDAC6-selective inhibition on the expression of the Th1-type T-cell polarizing transcription factors T-BET [29], and EOMES [30, 31]. T-BET expression was increased in ACY-1215 and ACY-241 treated T-cells (Additional file 2: Figure S4C). Paired analysis of ACY-1215 and ACY-241 treatments versus DMSO showed enhanced T-BET expression in CD8+ (*p* = 0.003, *p* = 0.001) and CD4+ (p = 0.019, *p* = 0.011) T-cells. We also found a modest but significant decrease in EOMES expression in CD8+ T-cells after treatment with ACY-1215 and ACY-241 (Additional file 2: Figure S4E; *p* = 0.037, *p* = 0.024).

### Expansion of T-cells in HDAC6 selective inhibitors reduced exhausted phenotypes

**Fig. 3:**
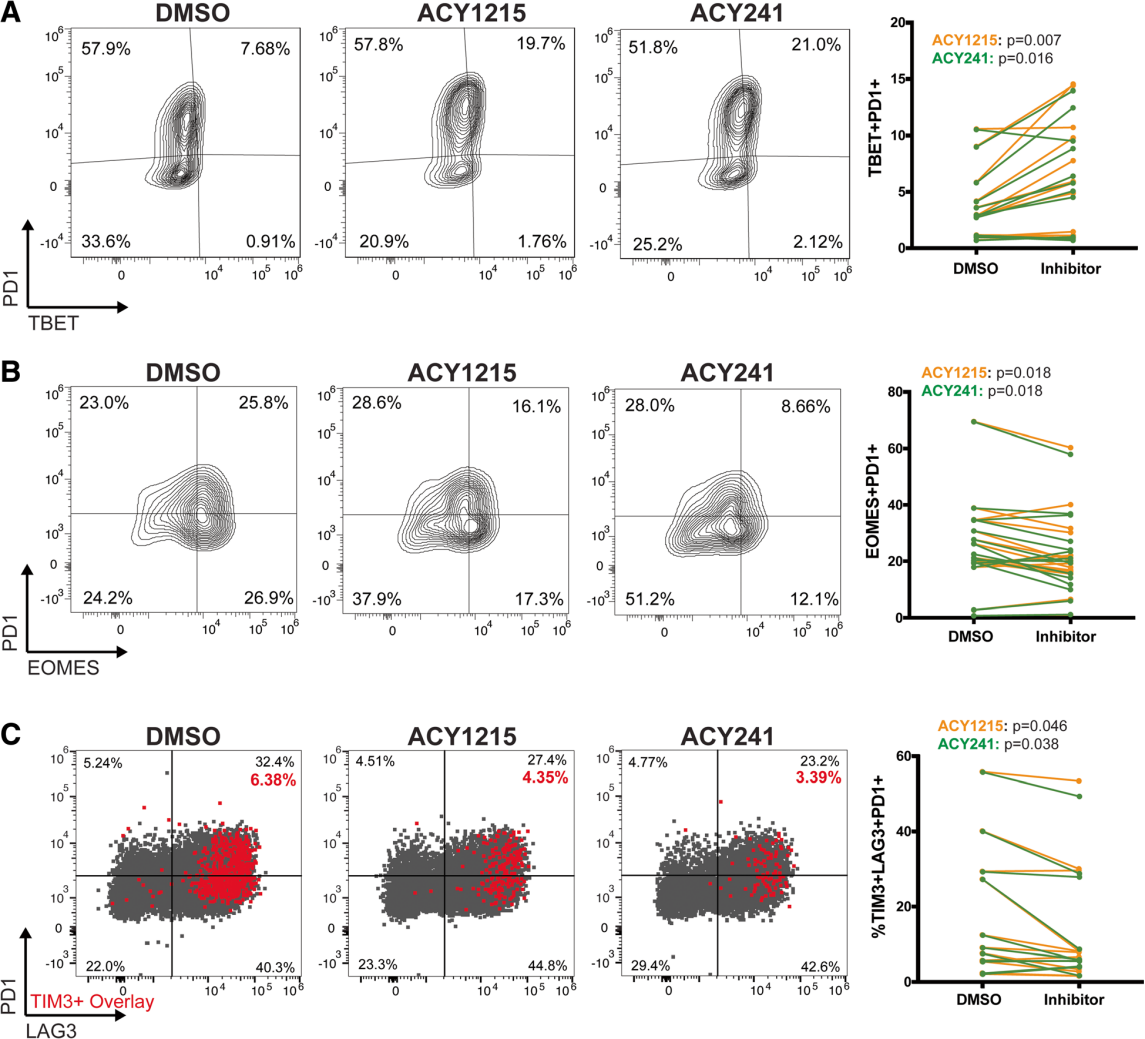
T-cells cultured with ACY-1215 or ACY-241 have reduced exhaustion associated phenotypes. T-cells from melanoma patient PBMCs were expanded with the indicated inhibitor (500nM), activated for 24 h, and evaluated by flow cytometry. Representative contour plots and paired analyses of percentages of CD8+ T-cells expressing (**a**) PD1 and TBET from twelve samples**,** (**b**) PD1 and EOMES from fifteen samples and (**c**) CD8+ T-cells expressing PD1, TIM3 and LAG3 from eleven samples are shown. Representative dot plots of PD1 vs LAG3 expression with TIM3+ cells overlaid in red are shown along with paired analyses (ACY-1215: orange lines, ACY-241 green lines). (**a**-**c**) Results shown are from over eleven patient samples assessed in three to five experiments. Each paired line represents an individual patient sample

Studies have shown that T-BET and EOMES expression by PD1-expressing T-cells are associated with effector function or exhaustion, respectively [30, 31]. We investigated changes in exhausted T-cell phenotypes after expansion with ACY-1215 or ACY-241. An increase in CD8 + TBET+PD1+ T-cells, a less terminally differentiated phenotype [30, 31], was found in T-cells expanded with ACY-1215 (*p* = 0.007) and ACY-241 (*p* = 0.016) (Fig. 3A). Conversely, ACY-1215 and ACY-241 reduced the proportion of EOMES+PD1+ T-cells (*p* = 0.018; Fig. 3B), a phenotype associated with terminal exhaustion [7, 30, 31]. ACY-1215 (*p* = 0.046) and ACY-241 (*p* = 0.038) reduced the percentage of T-cells co-expressing TIM3 + LAG3 + PD1+. Representative dot plots of LAG3 (x-axis) versus PD1 (y-axis) with TIM3-expressing cells overlaid in red along with paired analyses are shown in Fig. 3C. Although no changes were observed in PD1 as a single marker, expression of LAG3 (ACY-1215, *p* = 0.035; ACY-241, *p* = 0.009) and TIM3 (ACY-1215, p = 0.016; ACY-241, *p* = 0.005) were both decreased (Additional file 2 Figure S4F). In agreement with a reduction in exhaustion-associated phenotypes, we observed increased intracellular IFNγ and TNF in ACY-1215 (*p* = 0.0001) and ACY-241 (*p* = 0.001) treated and activated CD8+ T-cells (Additional file 2 Figure S4G). However, in cultures of total CD3+ T-cells expanded with ACY-1215/ACY-241 and activated prior to analysis, secreted levels of IFNγ and TNF were not significantly altered (Additional file 2: Figure S4H).

### Ex vivo expansion of T-cells in HDAC6 selective inhibitors increased the proportion of T-cells with central memory phenotypes

**Fig. 4:**
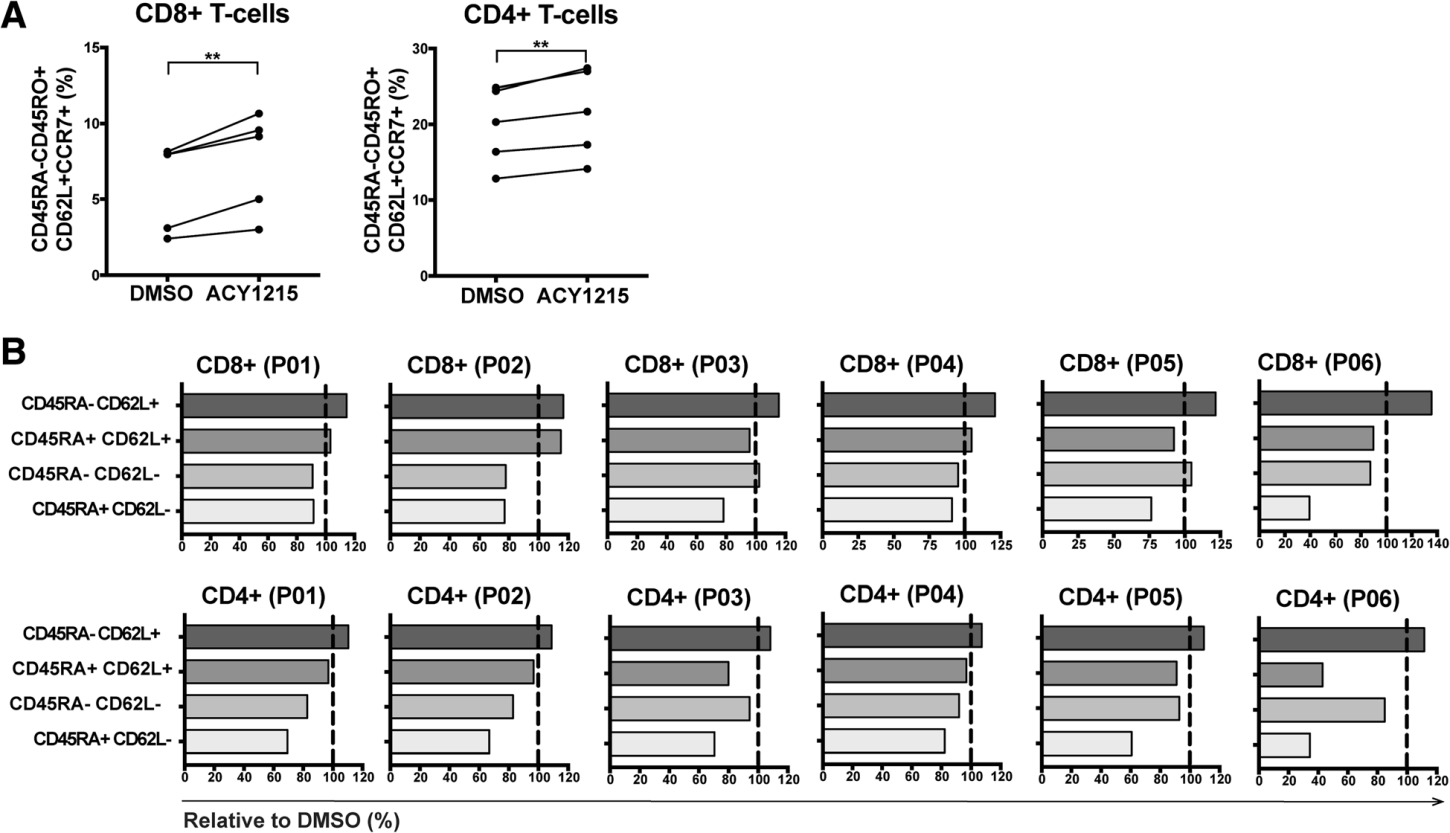
ACY-1215 increases the percentage of central memory T-cells. T-cells isolated from melanoma patient PBMC samples were expanded with DMSO or ACY-1215 500nM. (**a**) Paired analysis of CD45RA-CD45RO + CD62L + CCR7+ percentages after DMSO versus ACY-1215 treatment of five patient samples assessed. (**b**) CD8+ and CD4+ T-cells from six patients (P01–06) were assessed by flow cytometry for relative changes in the subsets: CD45RA-CD62L+ (central memory, Tcm), CD45RA + CD62L+ (Tnaïve), CD45RA-CD62L- (effector memory, Tem) and CD45RA + CD62L- (effector, Teff). Changes were calculated relative to DMSO, illustrated by the vertical black lines. (**a**-**b**) Samples were evaluated across three independent experiments. **p < 0.01

Given the role of T-BET in T-cell memory [32], we evaluated the ability of selective HDAC6 inhibitors to alter T-cell memory populations. T-cells from melanoma patient PBMCs were expanded for one week in 6000IU/mL IL-2, plus ACY-1215 or DMSO. CD8+ and CD4+ T-cells cultured with ACY-1215 displayed increased percentages of CD45RA-CD45RO + CD62L + CCR7+ central memory markers (*p* < 0.01) (Fig. 4A). The general flow cytometry gating strategy is shown in Additional file 2: Figure S5A. The frequencies of T-cells subsets after ACY-1215 treatment relative to DMSO control are graphed (Fig. 4B). In paired analyses (Additional file 2: Figure S5B), an increase in the proportion of CD45RA-CD62L+ (central memory phenotype) after HDAC6-selective inhibition was observed in CD8+ (top graphs) and CD4+ (bottom graphs) T-cells relative to DMSO (CD8+, *p* = 0.0033; CD4+: *p* = 0.0007). No consistent changes were observed in CD45RA + CD62L+ (naïve phenotype; *p* = 0.6006), or CD45RA-CD62L- (effector memory phenotype; *p* = 0.1353) CD8+ T-cells, while CD4+ T-cells had small but significant reductions in these populations (naïve: *p* = 0.0157; effector memory: *p* = 0.0016). Consistently decreased CD45RA + CD62L- (effector) frequencies were seen in CD4+ and CD8+ T-cells, trending towards reduction.

To determine whether the observed increase in central memory T-cells was independent of the high concentrations of IL-2 used (i.e. 6000IU/mL), T-cells were cultured for 72 h in lower dose IL-2 (100IU/mL) and treated with a range of concentrations (i.e. 10nM to 2uM) of ACY-1215 or ACY-241. T-cells were evaluated by flow cytometry for changes of CD62L + CD45RA- T-cells relative to DMSO. A dose-dependent increase in the proportion of central memory CD4+ and CD8+ T-cells was observed (Additional file 2: Figures S5C-D).

### Increased TIL central memory frequencies persisted after removal of selective HDAC6 inhibition

**Fig. 5:**
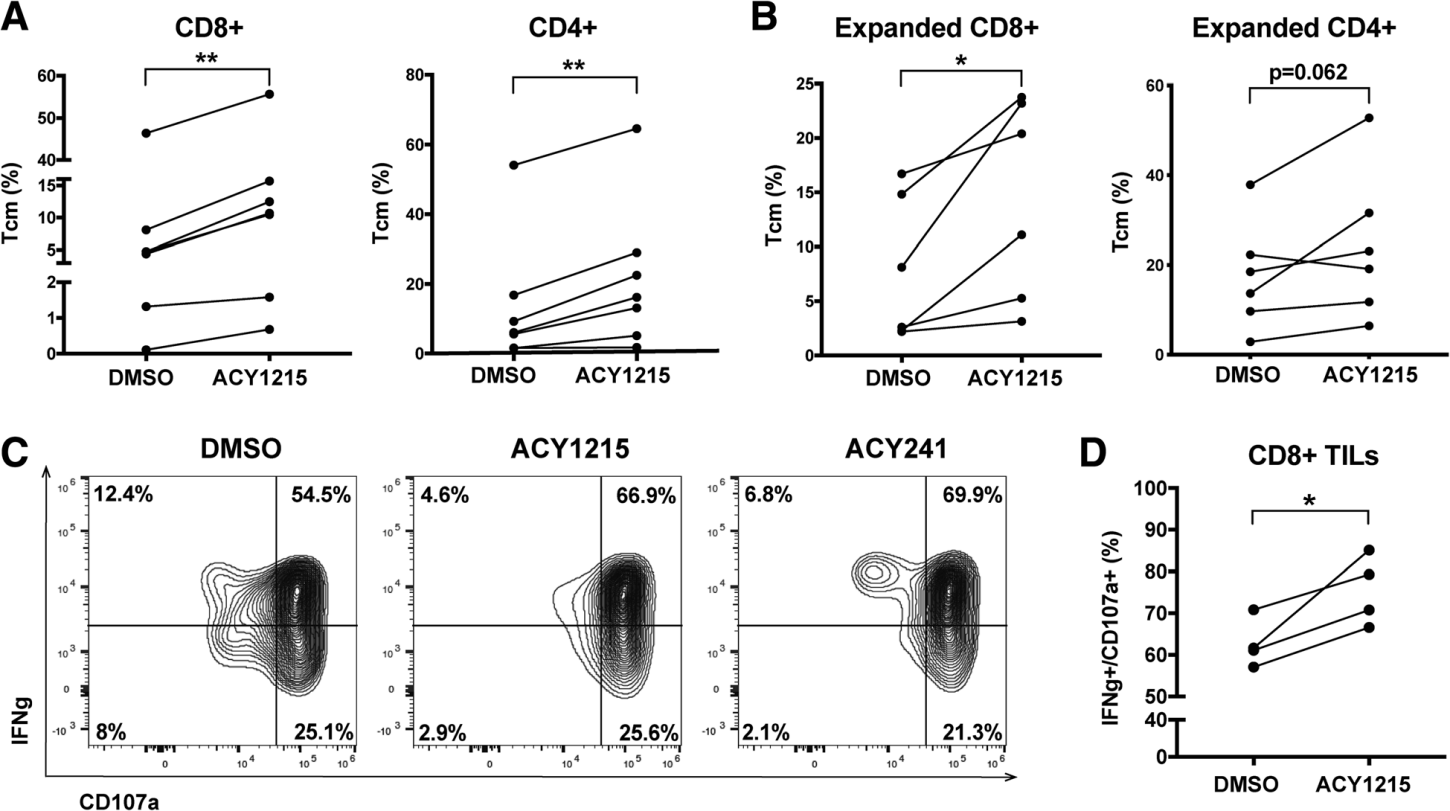
ACY-1215 and ACY-214 alter TIL phenotype. TILs harvested from resected melanoma tumors were expanded with 500nM ACY-1215 or DMSO. (**a**) CD8+ and CD4+ T-cells central memory percentages were assessed in six patient samples across four experiments based on CD45RO + CD62L+, CD45RA-CD62L+, CD45RA-CCR7+ or CD45RO + CD45RA-CD62L+ gating strategies. (**b**) After expansion, TILs were washed, activated and evaluated for the percent of CD45RO + CD62L+ or CD45RA-CD62L+ subsets. Data are from six patient samples assessed across four experiments. (**c**) TILs expanded with 500nM ACY-1215, 500nM ACY-241 or DMSO were stimulated with PMA/Ionomycin and monensin-treated for four hours. Intracellular IFNγ and CD107a expression was assessed by flow cytometry. Representative contour plots of CD8+ TILs from one out of four patient samples assessed are shown. (**d**) Paired analysis of DMSO versus ACY-1215 treated CD8+ IFNγ+CD107a + TILs are shown for all four patients assessed across three experiments. *p < 0.05

Since the presence of memory T-cells is associated with patient response to TIL adoptive cell therapy [33], we evaluated the ability of HDAC6-selective inhibitors to impact on the phenotype of TILs isolated from surgically resected melanoma tumors, by expanding TILs ex vivo in 6000IU/mL IL-2 [12], plus ACY-1215 or DMSO. Figure 5A shows an increase in central memory subsets in both CD8+ and CD4+ TILs (*p* = 0.0098, *p* = 0.0051) treated with ACY-1215 or DMSO. Percentages of naïve, central memory, effector and effector memory based on expression of CD45RA, CD45RO, CCR7 and/or CD62L are shown in Additional file 2: Figure S6 for CD8+ and CD4+ TILs harvested from patient tumors and grown ex vivo with ACY-1215 or DMSO. No significant differences in the number of TILs after expansion with DMSO versus ACY-1215 were observed (data not shown).

To determine the persistence of central memory markers in vitro after removal of the inhibitor, TILs were washed following expansion with ACY-1215 and further expanded via αCD3/CD28 activation for an additional week in the absence of ACY-1215. CD8+ TIL maintained an enhanced frequency of central memory phenotypes after ACY-1215 washout (*p* = 0.0177), while CD4+ TILs trended towards a similar increase (*p* = 0.062) (Fig. 5B).

### Expansion in the presence of HDAC6 selective inhibitors increased TIL cytolytic function

We next determined the functional impact of ACY-1215 on the cytolytic capacity of TILs by evaluating co-expression of CD107a and IFNγ after treatment. TILs were expanded for one week with ACY-1215 or ACY-241, then washed and activated. While 54.5% of DMSO-cultured TILs co-expressed CD107a and IFNγ, 66.9% of ACY-1215 and 69.9% of ACY-241 cultured TILs were positive for both markers (Fig. 5C). In paired analyses, TILs grown in ACY-1215 had similar increases in CD8 + CD107a + IFNγ+ T-cells (*p* = 0.0371) (Fig. 5D).

### T-cells expanded in the presence of HDAC6 selective inhibitors mediated enhanced target cell killing

**Fig. 6:**
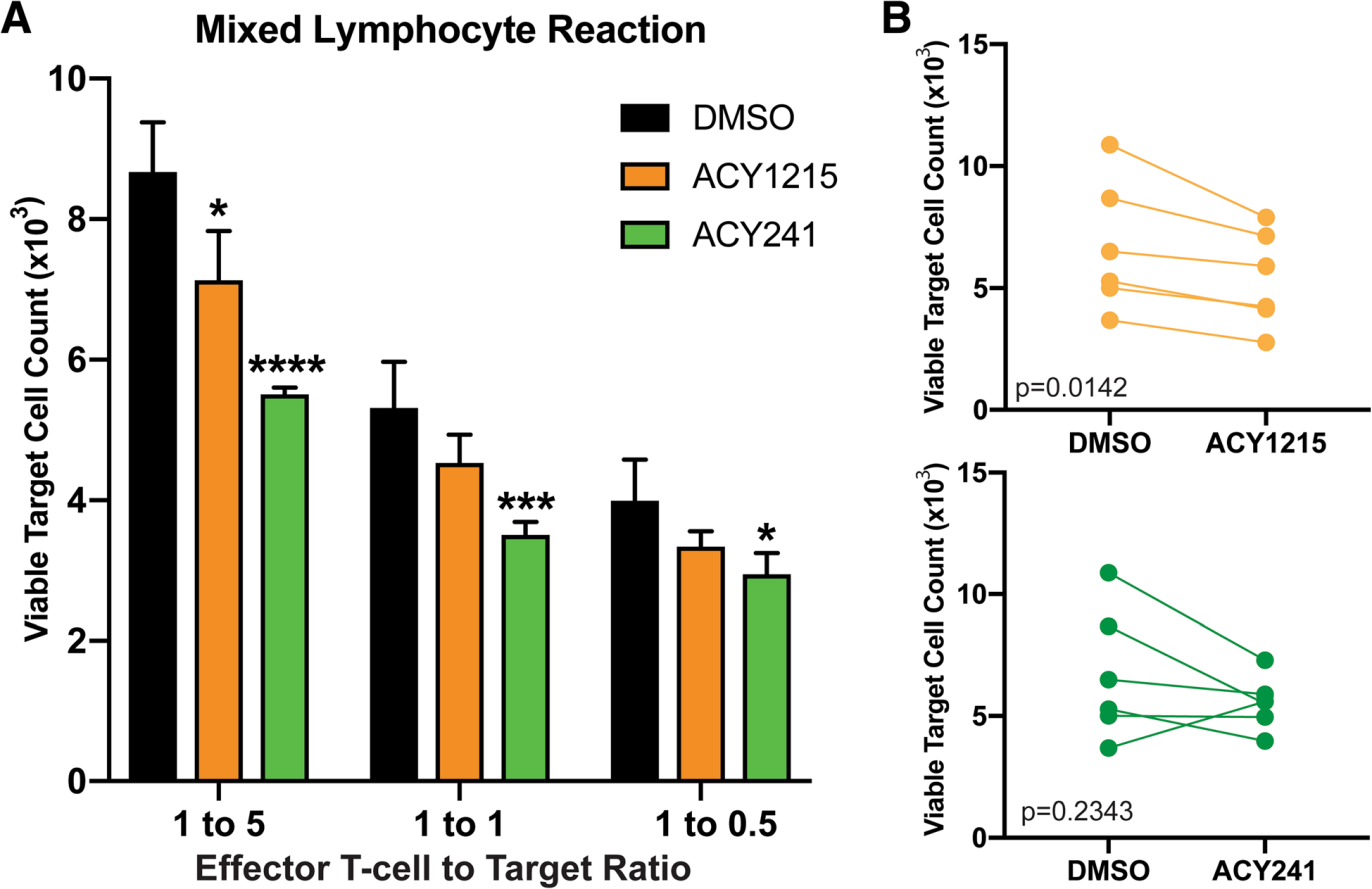
ACY-1215 Augments T-cell Cytotoxicity. T-cells from melanoma patient PBMC were expanded with DMSO, 500nM ACY-1215 or 500nM ACY-241, washed, then co-cultured with CFSE-labeled, irradiated allogeneic PBMC (target cells). (**a**) The numbers of viable target cells after co-culture are shown in a representative sample from one patient cultured at the indicated ratios (T-effector:Target) (triplicates assessed) and (**b**) in paired analyses of DMSO versus ACY-1215 and DMSO versus ACY-241 at 1:5 T-effector:Target ratio. Data are from six patient samples evaluated in two experiments. Error bars are +SEM. *p < 0.05, ***p < 0.001, ****p < 0.0001

We next determined if T-cells expanded with ACY-1215 or AC-Y241 had enhanced cytotoxic function. T-cells were expanded with 6000 IU/mL IL-2, plus ACY-1215 or ACY-241, washed, co-cultured with irradiated allogeneic PBMC (target cells) and assessed for the number of viable target cells by flow cytometry. Figure 6A shows that ACY-1215 and ACY-241 mediated enhanced target cell killing at the indicated ratios. Paired analysis of 1:5 T-effector:Target cell cultures demonstrates that ACY-1215-treated T-cells had increased killing (*p* = 0.0142), and though not statistically significant, ACY-241-treated T-cells had increased killing in 5/6 patient samples assessed (*p* = 0.2343) (Fig. 6B).

### ACY-1215 treatment altered the chromatin accessibility of T-cells

**Fig. 7:**
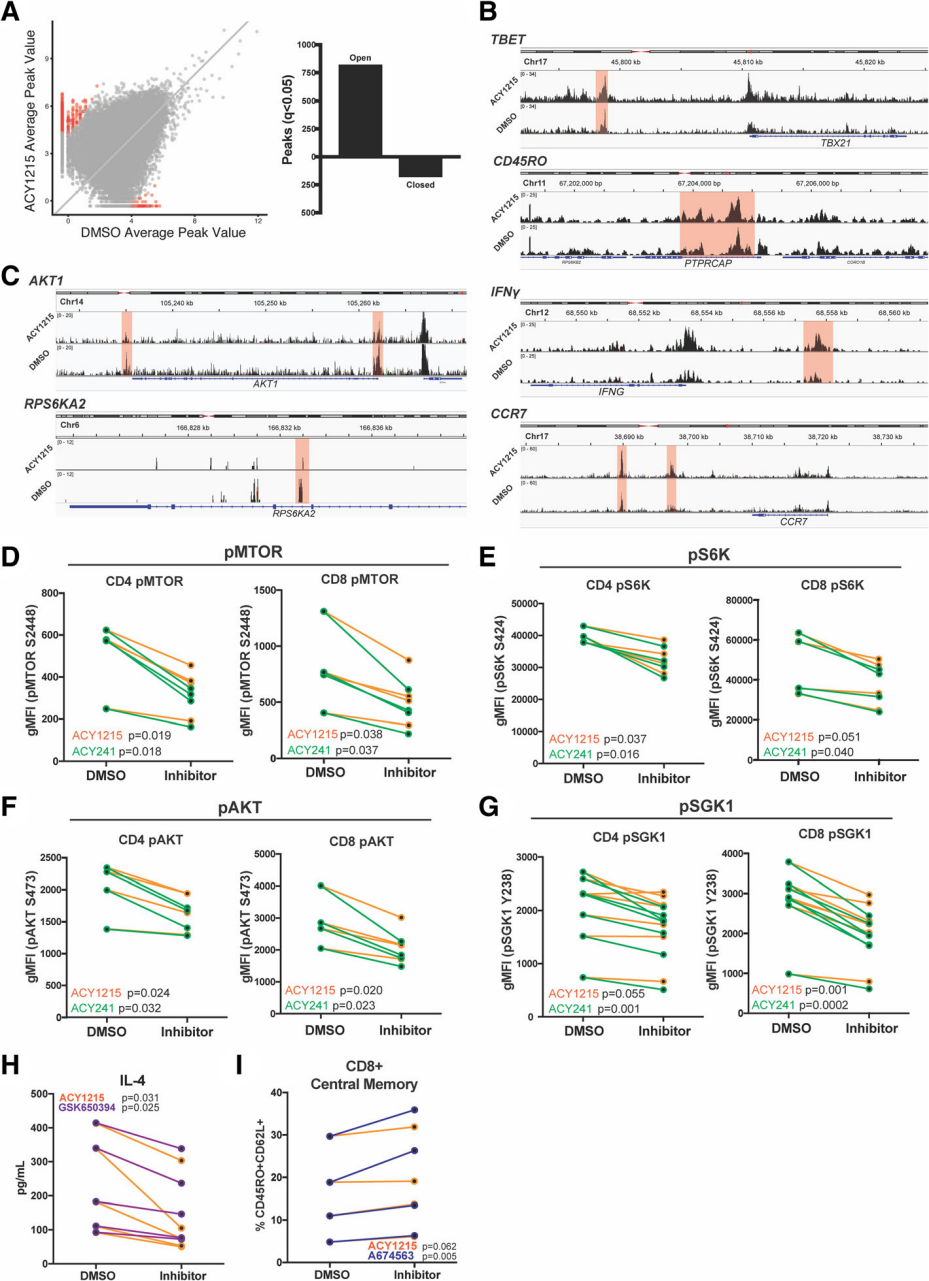
ACY-1215 alters chromatin accessibility of T-cells and downregulates mTOR signaling pathways. (**a**-**c**) ATAC-seq was performed in T-cells from three melanoma patient PBMCs following expansion with 500nM ACY-1215 or DMSO. (**a**) Scattered dot plot displaying significant peaks (red dots; q < 0.05) of open versus closed chromatin in ACY-1215-treated over DMSO control. Representative (**b**) open peaks in T-BET, CD45RO, IFNγ and CCR7 gene regions, and (**c**) closed peaks in AKT and S6K gene regions, in ACY-1215-treated over DMSO control. Significant regions are highlighted in red. All samples were adjusted for false discovery rate (FDR). Three patient samples were assessed. (**d**-**g**) After T-cell expansion with DMSO or HDACi, phosphorylation of (**d**) mTOR S2448, (**e**) S6K S424, (**f**) AKT S473 and (**g**) SGK1 Y238 in CD4+ and CD8+ T-cells was determined by flow cytometry and the geometric mean fluorescence (gMFI) graphed. At least four patient samples were assessed in two to three experiments for each phosphorylated marker evaluated. Each paired line represents an individual patient sample. (**h**) T-cells from five patient samples in two experiments were treated with GSK650394 100nM (SGK1 inhibitor), ACY-1215 500nM, or DMSO, activated and assessed for IL-4 secretion after 72 h. (**i**) CD8+ T-cells from four patient samples in two experiments were evaluated by flow cytometry for CD45RO + CD62L+ central memory percentage after expansion in A674563 50nM (AKT inhibitor) or ACY-1215 500nM. Comparisons were made against DMSO

To characterize potential epigenetic changes induced by ACY-1215, we evaluated chromatin accessibility using ATAC-Seq. T-cells were expanded with 6000IU/mL IL-2, plus ACY-1215 or DMSO. After adjusting for false discovery rate (FDR; q < 0.05), 812 chromatin regions (peaks) were found to be increased in accessibility (open) and 172 peaks were decreased in accessibility (closed) in ACY-1215 treated T-cells (Fig. 7A). Increases in chromatin accessibility in specific gene regions associated with phenotypic changes induced by ACY-1215 were found (Fig. 7B). These included increases in T-BET (q = 0.0196), two regions of CD45RO (q = 0.0196, q = 0.032), IFNγ (q = 0.0434) and two regions of CCR7 (q = 0.0196, q = 0.0342). Conversely, decreases in accessibility were seen in genes downstream of mTOR signaling (mTORC), including two regions of AKT1 (q = 0.0409, q = 0.0419), two regions of RPS6KA2 (q = 0.0419, q = 0.0446) (one illustrated region shown), and SGK1 (q = 0.048) (not illustrated) (Fig. 7C).

Pathways associated with significant peaks were evaluated using Enrichr [34]. Assessment using KEGG 2016 pathways of open peak-associated genes showed that TCR signaling was the pathway with the highest level of significance (FDR adjusted *p*-value = 0.01; Additional file 1 Table SII). To assess whether TCR signaling was also increased at a protein/functional level, T-cells were evaluated for phosphorylation of CD3ε after expansion and activation. Increases in CD3ε phosphorylation were observed in samples expanded with ACY-1215 (*p* = 0.0267) (Additional file 2: Figure S7A).

### HDAC6 selective inhibitors suppressed mTORC signaling in T-cells

mTORC1 and mTORC2 signaling pathways are associated with regulation of Th2 T-cell function [35], T-cell exhaustion [36], memory formation [37], and Treg formation and function [38]. Although we found that genes downstream of mTOR signaling were less accessible (Additional file 1: Table II), the function of these molecules at the protein level can only be determined by changes in phosphorylation. We assessed the effects of HDAC6-selective inhibition on mTOR signaling by expanding T-cells in 6000IU/mL IL-2, plus ACY-1215, ACY-241 or DMSO, and evaluating phosphorylation of mTOR downstream molecules by flow cytometry. As shown in Fig. 7D, decreased levels of phosphorylated mTOR were seen for both CD4+ and CD8+ T-cells treated with ACY-1215 (*p* < 0.038) and ACY-241 (*p* < 0.037). Reduced levels of downstream phospho-S6K (Fig. 7E; ACY-1215 *p* < 0.051, ACY-241 *p* < 0.04), phospho-AKT (Fig. 7F; ACY-1215 *p* < 0.024, ACY-241 *p* < 0.032), and phospho-SGK1 (Fig. 7G; ACY-1215 *p* < 0.055, ACY-241 *p* < 0.001) were also observed. Patient samples had decreased levels of phospho-mTOR in CD4 + CD25 + CD127- Tregs resulting from ACY-1215 (*p* = 0.0017) and ACY-241 treatments (*p* = 0.0002) (Additional file 2 Figure S7B).

### Specific inhibition of mTORC signaling molecules decreased IL-4 production and increased central memory T-cells

We evaluated whether mTORC-specific inhibitors could recapitulate the ACY-1215/ACY-241 effects on T-cells. T-cells were treated with ACY-1215 or the SGK1 inhibitor GSK650394 [39], and subsequently activated via αCD3/CD28. As was seen previously in Fig. 2A, Fig. 7H shows that ACY-1215 treatment reduced production of IL-4 (*p* = 0.031). SGK1 specific inhibition also resulted in decreased IL-4 production (*p* = 0.025) with no changes in IFNγ production (data not shown).

AKT inhibition has previously been shown to promote expansion of T-cells with a memory phenotype [37]. To determine whether AKT inhibition recapitulated the increase in central memory T-cells observed with HDAC6-selective inhibition, T-cells were expanded with ACY-1215 or the AKT inhibitor A674563 [40]. Cultures were assessed for a central memory phenotype. As expected, ACY-1215 treatment increased the percentages of CD8 + CD45RO + CD62L+ T-cells (*p* = 0.062). AKT inhibition resulted in a similar increase in CD8 + CD45RO + CD62L+ T-cells (*p* = 0.005) (Fig. 7I).

## Discussion

Our results demonstrate that the HDAC6-selective inhibitors ACY-1215 and ACY-241 alter melanoma patient T-cell phenotypes and function ex vivo at concentrations achievable in human patients [41], resulting in enhanced effector function without impacting viability. While both class I (MS275) and HDAC6-specific (Tubastatin A) inhibitors altered Th2 cytokine secretion, ACY-1215 and ACY-241 reduced production of all Th2 cytokines evaluated. In contrast to ACY-1215 and ACY-241, class I HDAC inhibition resulted in increased IL-10 expression. HDAC6 inhibition has previously been shown to downregulate expression of IL-10 [42], while pan-HDAC inhibition has been shown to upregulate IL-10 production in immune cells [43]. It is important to note that the role of IL-10 in cancer immunotherapy is nuanced, with IL-10 being associated with detrimental and beneficial effects dependent on context [44]. Different concentrations of inhibitors may also produce distinct cytokine secretion profiles. However, concentrations >1μM of class I HDAC and HDAC6-selective inhibitors and > 10nM of the pan-HDAC inhibitor LBH589 reduced T-cell viability. These results suggest that maximizing tumor cytotoxicity using HDAC inhibitors may be counteracted by deleterious effects on immune cell function and viability.

Reduction of Th2 cytokine levels induced by HDAC6-selective inhibition was accompanied by decreased GATA3 and increased T-BET transcription factor expression. GATA3 drives T-cells towards a Th2 response while suppressing T-BET [45]. ACY-1215 and ACY-241 treatments led to downregulation of phosphorylated SGK1, a known regulator of Th2 polarization and directly downstream of mTORC2 [35]. Inhibition of SGK1 recapitulated the decreased Th2 cytokine secretion seen with HDAC6-selective inhibitors, suggesting that alteration in mTORC signaling by HDAC6 selective inhibitors may contribute to the observed decrease. While beyond the scope of this study, it is possible that ACY-1215/ACY-241 directly modulate acetylation of unidentified substrates of HDAC6 that interfere with mTORC signaling.

ACY-1215 and ACY-241 treatments hindered Treg suppression. Research has shown that abrogation of mTORC1 downstream signaling reduced Treg suppressive function [38], similar to the effects with HDAC6 selective inhibition reported in this study. ACY-1215 and ACY-241 also downregulated FOXP3 expression at protein and mRNA levels. While the class I inhibitor MS275 did significantly downregulate FOXP3 protein expression as well, the degree was minimal. Combining a class I inhibitor with an HDAC6-specific inhibitor did not recapitulate the effects seen with ACY-1215/ACY-241 to the same magnitude. This may be attributed to differences in potency or the stochastic nature of HDAC inhibition. Interestingly, acetylation of histones associated with several transcription factor binding regions of the *foxp3* gene were upregulated after treatment with ACY-1215. SMAD3 and RUNX3 are known promoters of *foxp3* [46, 47], and increased histone acetylation of their binding sites on the *foxp3* gene are suggestive of increased expression. However, ACY-1215 downregulated *foxp3* at the mRNA level. This may be partially attributable to a concomitant increase in histone acetylation of the GATA3 binding region of *foxp3*, a known negative regulator [48]. To further complicate the interpretation of these data, GATA3 was downregulated in ACY-1215-treated cells, making its ability to potently downregulate *foxp3* expression questionable. While beyond the scope of this manuscript, these results reflect a highly complex interplay regulating FOXP3 expression.

In contrast to the observed phenotypes resulting from Treg treatment with ACY-1215 and ACY-241, genetic abrogation of HDAC6 and its specific inhibition were previously shown to result in a more suppressive Treg phenotype, with enhanced FOXP3 expression [20]. However, decreased Treg frequencies and FOXP3 expression upon treatment with HDAC6-selective inhibitors have also been demonstrated in models of non-small cell lung cancer [22] and multiple myeloma [23]. This discrepancy likely results from ACY-241 and ACY-1215 targeting both HDAC6 and class I HDACs at the concentrations used, as previous studies have shown that class I HDAC inhibition results in a similar Treg phenotype to that observed with ACY-1215 and ACY-241 treatment [21]. Comparatively, ACY-1215 and ACY-241 reduced iTreg suppressive function seemingly more robustly than with nTregs, particularly melanoma patient-derived nTregs. We hypothesize that this is due to increased phenotypic plasticity of naïve CD4+ T-cells relative to lineage committed nTregs.

ACY-1215 and ACY-241 reduced populations of T-cells co-expressing TIM3, LAG3 and PD1 as well as EOMES and PD1, suggesting that these compounds decreased T-cell exhaustion phenotypes and inhibitory functions. T-cells expanded with ACY-1215 and ACY-241 displayed increased percentages of TBET+PD1+ T cells. In contrast to EOMES+PD1+ T-cells, TBET+PD1+ T-cells are responsive to checkpoint blockade immunotherapy [30]. The phenotypic shift observed lends support to combining HDAC6-selective inhibitors with checkpoint blockade therapies.

In contrast to reducing T-cell immunosuppressive phenotypes, HDAC6-selective inhibition enhanced the proportion of central memory T-cells, a subset associated with improved patient outcome and survival after TIL adoptive cell therapy [33]. While 500nM ACY-1215/ACY-241 concentrations consistently increased central memory percentages, the increases were most pronounced at concentrations >1uM, but at the expense of T-cell viability. mTORC signaling pathways can increase memory T-cells [49], with AKT inhibition enhancing CD62L expression, T-cell central memory properties [37], and reducing terminal differentiation [50]. Consistent with those findings, HDAC6-selective inhibition decreased chromatin accessibility at the AKT gene region and reduced phosphorylated-AKT at the protein level. T-cell treatment with an AKT inhibitor enhanced accumulation of CD45RO + CD62L+ T-cells, suggesting that AKT downregulation by ACY-1215 and ACY-241 may be partially involved in maintaining a central memory phenotype.

An increased proportion of central memory cells was also seen in patient TIL samples expanded with HDAC6-selective inhibitors, which was maintained even after drug removal and continued expansion. CD8+ TILs grown in ACY-1215 and ACY-241 demonstrated higher production of the cytolytic markers CD107a and IFNγ upon activation. CD8+ T-cells derived from PBMC also expressed increased intracellular IFNγ and TNF upon ACY-1215/ACY-241 treatments and activation. However, such increase was not observed in Th1 cytokines secreted by CD3+ T-cells, suggesting that these compounds may be altering cytokine production in specific cell subsets and/or not substantially enough to impact total secreted levels. Indeed, increased open chromatin peaks in IFNγ gene regions after ACY-1215 treatment was not accompanied by higher levels of secreted IFNγ by T-cells, highlighting that studies investigating the effects and mechanisms of HDAC6-selective inhibitors in specific T-cell subpopulations should be further explored.

Collectively, these results indicate that ACY-1215 and ACY-241 impact T-cells from melanoma patients by downregulating Th2 cytokine production and Treg expansion/function, as well as decreasing T-cell exhaustion phenotypes, while augmenting the proportion of central memory cells, expression of cytolytic markers and T-cell killing ability in vitro. In agreement with a recent study by Bae et al. [23], these results support a model in which HDAC6-selective inhibition downregulates mTORC1/2 signaling thereby, in part, altering T-cell function. Although ACY-1215 and ACY-241 improved melanoma patient immune effector function in vitro, further studies evaluating anti-tumor therapeutic efficacy in tumor models in vivo and in cancer patients are warranted.

## Additional files

Additional file 1: **Table S1.** Flow 1 cytometry antibodies. **Table S2.** Pathway analyses based on Enrichr assessment 1 and KEGG 2106. (PDF 105 kb)

Additional file 2: **Figure S1.** Impact of HDAC inhibitors on protein acetylation. **Figure S2.** Changes in Treg phenotype and function induced by HDAC inhibition. **Figure S3.** Effects of HDAC inhibitors on T-cell cytokine production. **Figure S4.** ACY-1215 and ACY-241 Effects on Transcription Factor 1 Expression and T-cell. **Figure S5.** Changes in T-cell memory phenotype induced by HDAC 1 inhibition. **Figure S6.** Changes in TIL subsets following expansion with HDAC inhibitors. **Figure S7.** ACY-1215 and ACY-241 effects on T-cell signaling. (PDF 2705 kb)

## Abbreviations

FDR: false discovery rate
HDAC: Histone deacetylase
HDACi: HDAC inhibitor
iTreg: inducible Treg
nTreg: natural Treg
Tcon: conventional T-cell
Treg: regulatory T-cells
