# Supplementary material for: HDAC6 selective inhibition of melanoma patient T-cells augments anti-tumor characteristics
Source: J Immunother Cancer. 2019 Feb 6;7:33. doi: 10.1186/s40425-019-0517-0 (PMC6366050; doi:10.1186/s40425-019-0517-0)
Supplement: Supplementary file 1 — Table S1. Flow 1 cytometry antibodies. Table S2. Pathway analyses based on Enrichr assessment 1 and KEGG 2106. (PDF 105 kb) [file 40425_2019_517_MOESM1_ESM.pdf]

Supplemental Table 1. Flow cytometry antibodies.

Marker	Clone	Vendor
CD3	HIT3a	BD Bioscience
CD3	SK7	BD Bioscience
CD3	UCHT1	BD Bioscience
CD3	UCHT1	eBioscience
CD3	UCHT1	BioLegend
CD4	RPA-T4	BD Bioscience
CD8	RPA-T8	BD Bioscience
CD127	HIL-7R-M21	BD Bioscience
CD25	M-A251	BD Bioscience
CD25	2A3	BD Bioscience
FOXP3	236A/E7	BD Bioscience
FOXP3	259D/C7	BD Bioscience
Ki67	B56	BD Bioscience
Ki67	20Raj1	eBioscience
GATA3	L50-823	BD Bioscience
CD279 (PD1)	MIH4	BD Bioscience
CD279 (PD1)	MIH4	eBioscience
TBET	O4-46	BD Bioscience
TBET	eBio4B10	eBioscience
EOMES	WD1928	eBioscience
CD223 (LAG3)	3DS223H	eBioscience
CD223 (LAG3)	3DS223H	BioLegend
CD366 (TIM3)	F38-2E2	eBioscience
CD366 (TIM3)	F38-2E2	BioLegend
CD45RA	HI100	BD Bioscience
CD45RA	HI100	eBioscience
CD45RO	UCHL1	BD Bioscience
CD62L	DREG-56	BD Bioscience
CD62L	DREG-56	eBioscience
CD197 (CCR7)	3D2	BD Bioscience
IFNγ	B27	BD Bioscience
IFNγ	4S.B3	BD Bioscience
CD107a	H4A3	BD Bioscience
phospho-mTOR (S2448)	O21-404	BD Bioscience
phospho-S6K (S424)	ab47379	Abcam
phospho-AKT (S473)	M89-61	BD Bioscience
phospho-SGK1 (Y238)	Y238	Abcam
acetyl-αTUBULIN (Lys40)	D20G3	Cell Signaling
acetyl-HISTONE 3 (Lys9, Lys14)	HIST2H3C	NovusBio

Supplemental Table 2. Pathway analyses based on Enrichr assessment and KEGG 2106.

KEGG 2016 Open	Overlap	P-value	Adjusted P-value	Z-score	Combined Score	Genes
T cell receptor signaling pathway_Homo sapiens_hsa04660	16/104	0.000040173	0.010003017	-1.881013558	19.04022375	ITK; NFATC2; NFATC1; PIK3R1; MAPK14; CD3D; VAV1; CDC42; PTPRC; IFNG; CD8A; GRAP2; MAP3K8; GRB2; LCP2; PAK4
AGE-RAGE signaling pathway in diabetic complications_Homo sapiens_hsa04933	15/101	0.000104769	0.013043711	-2.033602679	18.63543649	SMAD4; SMAD3; VEGFB; PRKCA; NFATC1; PIK3R1; MAPK14; TGFBR1; CDC42; DIAPH1; CCND1; PLCG2; BCL2; BAX; JAK2
Ras signaling pathway_Homo sapiens_hsa04014	24/227	0.000295537	0.022391294	-1.956844824	15.90272208	PRKCG; PLA2G2F; NGFR; KSR1; PLA2G2C; INSR; RASGRF2; PDGFB; VEGFB; PRKCA; FASLG; PIK3R1; ETS1; CDC42; EFNA3; GNG2; RASA3; RASSF5; PLCG2; REL; GRB2; FGFR4; RGL2; PAK4
HTLV-I infection_Homo sapiens_hsa05166	26/258	0.000359699	0.022391294	-1.868059228	14.81416098	CD40; PDGFB; SLC2A1; PIK3R1; ITGAL; ADCY7; CD3D; ETS1; CCND1; CDC27; BUB3; HLA-DPA1; IL15RA; SMAD4; SMAD3; CDKN2A; NFATC2; NFATC1; WNT9A; TGFBR1; CREB1; CDC16; BAX; ANAPC5; TLN1; ATF3
Leukocyte transendothelial migration_Homo sapiens_hsa04670	15/118	0.000592369	0.029499985	-1.623625014	12.06577527	PRKCG; ITK; MSN; PRKCA; PIK3R1; MAPK14; F11R; ITGAL; VAV1; GNAI2; CDC42; RASSF5; PLCG2; PTK2B; SIPA1
Hepatitis B_Homo sapiens_hsa05161	17/146	0.000736597	0.0305688	-1.808920364	13.04859068	PRKCG; SMAD4; SMAD3; DDX3X; NFATC2; PRKCA; FASLG; NFATC1; PIK3R1; TGFBR1; CREB1; CASP8; CCND1; BCL2; BAX; PTK2B; GRB2
Non-small cell lung cancer_Homo sapiens_hsa05223	9/56	0.001399224	0.045935991	-1.857769395	12.20895815	PRKCG; RXRA; CCND1; CDKN2A; RASSF5; PLCG2; PRKCA; GRB2; PIK3R1
Pathways in cancer_Homo sapiens_hsa05200	33/397	0.001930783	0.045935991	-1.869538519	11.68429723	PDGFB; SLC2A1; FASLG; PIK3R1; ADCY7; ETS1; GNAI2; CDC42; GNG2; RXRA; CASP8; CCND1; RASSF5; PLCG2; TCEB1; BID; PRKCG; SMAD4; SMAD3; CDKN2A; TPM3; PTCH1; DAPK2; VEGFB; PRKCA; TRAF2; WNT9A; TGFBR1; RUNX1; MSH3; BCL2; BAX; GRB2
Rap1 signaling pathway_Homo sapiens_hsa04015	21/211	0.001487683	0.045935991	-1.785469563	11.62436318	PRKCG; MAP2K3; NGFR; INSR; PDGFB; VEGFB; ARAP3; PRKCA; PIK3R1; MAPK14; ITGAL; ADCY7; GNAI2; CDC42; EFNA3; RASSF5; LCP2; TLN1; FGFR4; SKAP1; SIPA1
Osteoclast differentiation_Homo sapiens_hsa04380	15/132	0.001882809	0.045935991	-1.657093966	10.39824858	IFNAR2; NFATC2; TRAF2; NFATC1; PIK3R1; MAPK14; TGFBR1; CYLD; CREB1; IFNG; CTSK; PLCG2; GRB2; LCP2; JUNB

KEGG 2016 Close	Overlap	P-value	Adjusted P-value	Z-score	Combined Score	Genes
mTOR signaling pathway_Homo sapiens_hsa04150	4/60	0.003827604	0.563083199	-1.985962219	11.05290507	IKBKB; RPS6KA2; AKT1; PRKCA
B cell receptor signaling pathway_Homo sapiens_hsa04662	4/73	0.007677239	0.563083199	-1.963923608	9.563316666	IKBKB; CD19; AKT1; NFATC1
Osteoclast differentiation_Homo sapiens_hsa04380	5/132	0.013600716	0.563083199	-1.812386465	7.788971538	IKBKB; IFNGR1; AKT1; NFATC1; SQSTM1
Natural killer cell mediated cytotoxicity_Homo sapiens_hsa04650	5/135	0.014863311	0.563083199	-1.770765414	7.452902785	NCR2; IFNGR1; PRKCA; NFATC1; HLA-A
Vibrio cholerae infection_Homo sapiens_hsa05110	3/51	0.01704845	0.563083199	-1.793071285	7.30084112	MUC2; ATP6V1H; PRKCA
AGE-RAGE signaling pathway in diabetic complications_Homo sapiens_hsa04933	4/101	0.022916177	0.563083199	-1.890556431	7.138575114	AKT1; PRKCA; NFATC1; PRKCZ
Acute myeloid leukemia_Homo sapiens_hsa05221	3/57	0.022872176	0.563083199	-1.835519219	6.934287151	IKBKB; AKT1; RUNX1
Insulin resistance_Homo sapiens_hsa04931	4/109	0.029264773	0.600638482	-1.675947034	5.918390383	IKBKB; RPS6KA2; AKT1; PRKCZ
Epithelial cell signaling in Helicobacter pylori infection_Homo sapiens_hsa05120	3/68	0.036007798	0.600638482	-1.663268685	5.528737957	IKBKB; PTPRZ1; ATP6V1H
Neurotrophin signaling pathway_Homo sapiens_hsa04722	4/120	0.0395401	0.600638482	-1.646061487	5.317502753	IKBKB; RPS6KA2; GAB1; AKT1
