# Supplementary material for: HDAC6 selective inhibition of melanoma patient T-cells augments anti-tumor characteristics
Source: J Immunother Cancer. 2019 Feb 6;7:33. doi: 10.1186/s40425-019-0517-0 (PMC6366050; doi:10.1186/s40425-019-0517-0)
Supplement: Supplementary file 2 — Figure S1. Impact of HDAC inhibitors on protein acetylation. Figure S2. Changes in Treg phenotype and function induced by HDAC inhibition. Figure S3. Effects of HDAC inhibitors on T-cell cytokine production. Figure S4. ACY-1215 and ACY-241 Effects on Transcription Factor 1 Expression and T-cell. Figure S5. Changes in T-cell memory phenotype induced by HDAC 1 inhibition. Figure S6. Changes in TIL subsets following expansion with HDAC inhibitors. Figure S7. ACY-1215 and ACY-241 effects on T-cell signaling. (PDF 2705 kb) [file 40425_2019_517_MOESM2_ESM.pdf]

Supplemental Figure 1.

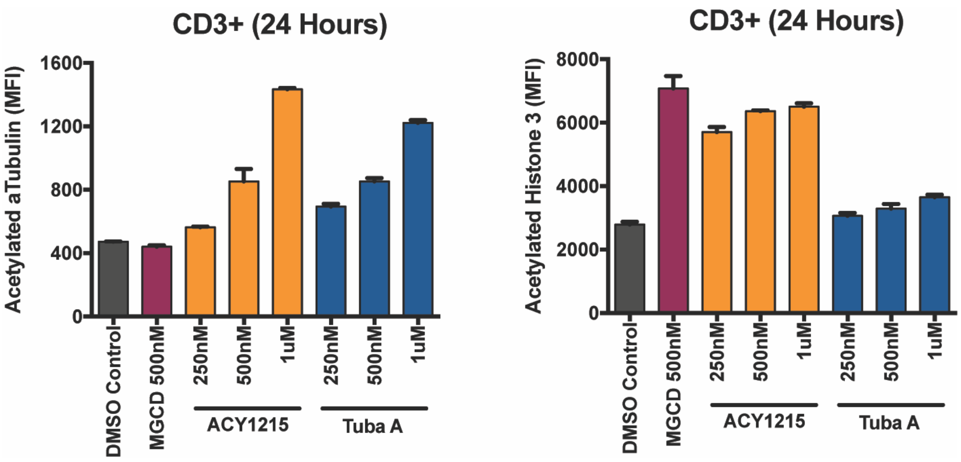

Supplemental Figure 1. Impact of HDAC inhibitors on protein acetylation. PBMC from melanoma patients were cultured with the indicated inhibitor and concentration for 24 hours. Levels of acetylated α-tubulin and acetylated histone 3 were assessed in CD3+ T-cells by flow cytometry. Representative data from one patient is shown; two independent patient samples were evaluated.

Supplemental Figure 2.

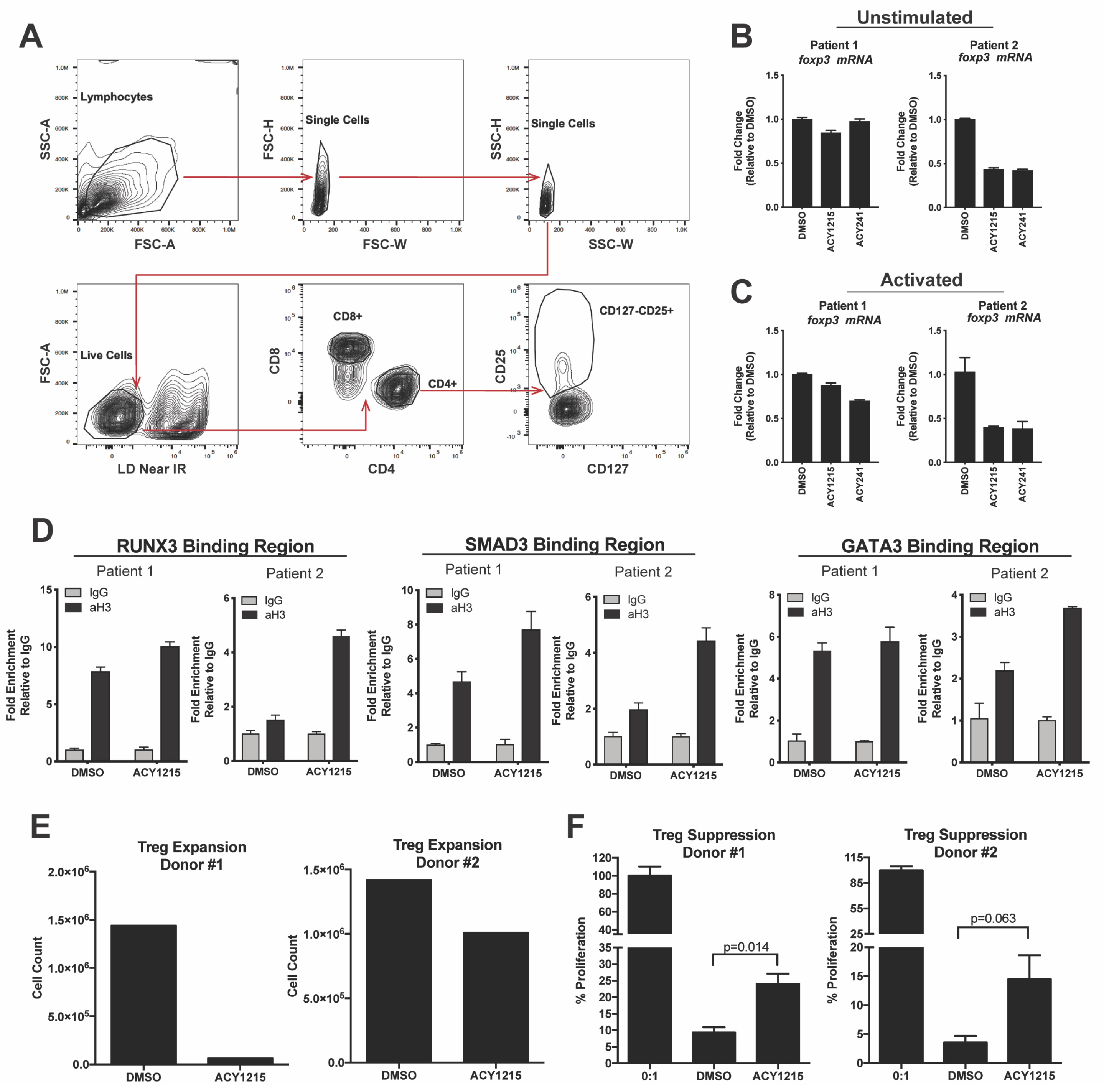

Supplemental Figure 2. Changes in Treg phenotype and function induced by HDAC inhibition. (A) The general gating strategy for analysis of Tregs in flow cytometry experiments is shown. **(B-C)** CD4+ T-cells from two melanoma patient samples were treated with DMSO, ACY-1215 500nM or ACY-241 500nM and 100IU/mL IL-2 for 24 hours. Cells were left **(B)** unstimulated or **(C)** activated, and mRNA *foxp3* expression was assessed

by qRT-PCR, as described in Woods DM *et al.*[1] 18S ribosomal RNA was used as reference gene (18S: Fw 5'-GTAACCCGTTGAACCCCATT-3', Rv 5'-CCATCCA- ATCGGTAGTAGCG-3') and the QuantiTect foxp3 Primer set (Qiagen; Venlo, Netherlands) was used to determine foxp3 mRNA expression. Error bars are representative of technical replicates. **(D)** CD3+ T-cells from two melanoma patient samples were treated with DMSO or ACY-1215 500nM and activated with Dynabeads. Cells were fixed after 24 hours and lysed using the ChIP-IT PBMC kit (Active Motif; Carlsbad, CA), per manufacturer's instructions. ChIP for acetylated histone 3 (ActiveMotif; aH3, black bars) and IgG isotype (Abcam; grey bars) were performed as described in Woods DM *et al*.[1] Fold enrichment over corresponding IgG DNA pulldown at RUNX3, SMAD3 and GATA3 binding regions were determined by qRT-PCR. Primers sequences were previously described[2] and are as follows: RUNX3_441 Fw: 5'-GTGGTGAGGGGAAGAAATCATA-3', Rv: 5'-GATGAGTGTGTGCGCTGATAA-3'. CNS1_SMAD3 Fw: 5'-AGGTTAAGAGTGTGGGTACTGG-3', Rv: 5'-TGAGGAAATGGAGGTATGGA-3'. CNS2_GATA3 Fw: 5'-GGACATCACCTACCACATCC-3', Rv: 5'-ACCACGGAGGAAGAGAAGAG-3'. Error bars are representative of technical replicates. **(E-F)** CD4+CD127-CD25+ nTregs were isolated from two healthy donor leukapheresis by magnetic bead purification (Miltenyi Biotec; Bergisch-Gladbach, Germany), as previously described[3]. Monocyte dendritic cells (moDC) were generated from healthy blood donors using IL-4 (1,000IU/ml) and GM-CSF (1,000IU/ml). The allogeneic moDC were irradiated (3,000 cGy) and cultured with the nTregs at a ratio of 1:1 for a total of 12 days with ACY-1215 at 500nM or DMSO equivalent. The culture medium was supplemented with IL-2 (10 IU/ml) and IL-15 (10ng/ml). A limiting dilution analysis was performed on day 0 and day twelve of culture to determine the frequency of moDC-expanded, antigen-specific nTreg. The frequency was calculated as previously described[3]. Expanded ACY-1215- or DMSO-treated nTreg were harvested and tested for suppressive potency in standard mixed lymphocyte reactions. T effectors (CD4+, CD25-) were cultured with fresh moDCs from the original stimulator at a ratio of 1:10. ACY-1215- or DMSO-treated nTreg (autologous to the T-effector and allogeneic to the moDCs) were mixed with the cultures at a Treg to T-effector ratio of 1:500. No additional ACY-1215 or DMSO was added to these cultures. Proliferation was measured by 3H-thymidine incorporation after 6 days. **(E)** Cell counts of ACY-1215 500nM or DMSO-treated nTregs were assessed twelve days after expansion. **(F)** Tcons were co-cultured with Tregs previously expanded in DMSO or ACY-1215 for twelve days and evaluated for proliferation.

1 Woods, D. M. *et al.* HDAC Inhibition Upregulates PD-1 Ligands in Melanoma and Augments Immunotherapy with PD-1 Blockade. *Cancer immunology research* **3**, 1375-1385, doi:10.1158/2326-6066.CIR-15-0077-T (2015).

2 Chakraborty, S. *et al.* Transcriptional regulation of FOXP3 requires integrated activation of both promoter and CNS regions in tumor-induced CD8(+) Treg cells. *Scientific reports* **7**, 1628, doi:10.1038/s41598-017-01788-z (2017).

3 Veerapathran, A. *et al.* Human regulatory T cells against minor histocompatibility antigens: ex vivo expansion for prevention of graft-versus-host disease. *Blood* **122**, 2251-2261, doi:10.1182/blood-2013-03-492397 (2013).

Supplemental Figure 3.

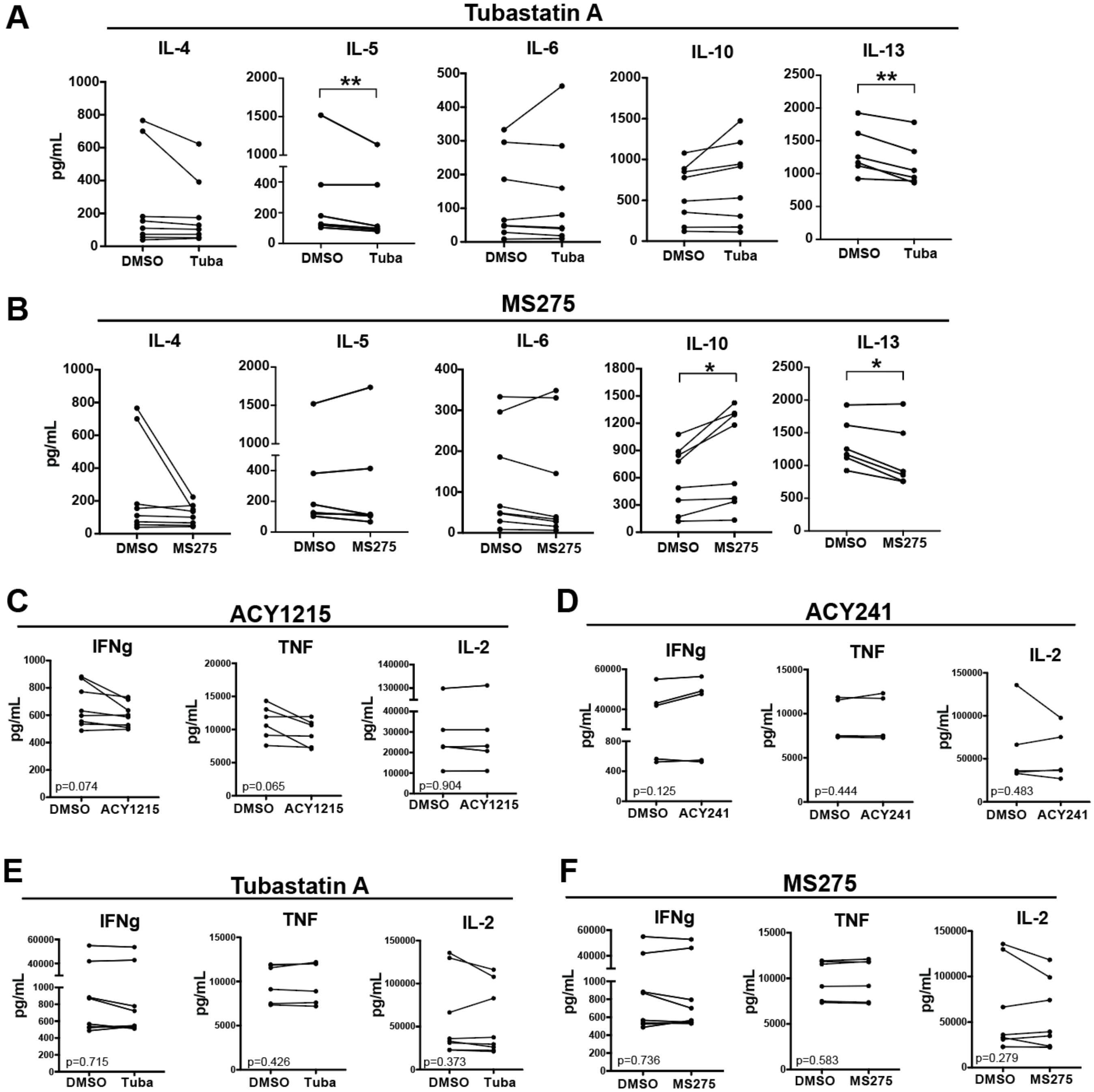

Supplemental Figure 3. Effects of HDAC inhibitors on T-cell cytokine production. CD3+ T-cells from melanoma patient PBMCs were treated with DMSO or HDAC inhibitors and activated for 72 hours. Cytokine secretion was assessed by Luminex. Paired analysis of IL-4, IL-5, IL-6, IL-10 and IL-13 production after **(A)** Tubastatin A 1μM and **(B)** MS275 125nM treatments are graphed. Paired analysis of IFNγ, TNF and IL-2 levels after **(C)** ACY-1215 500nM, **(D)** ACY-241 500nM, **(E)** Tubastatin A 1μM and **(F)** MS275 125nM treatments are shown. Results are shown for at least six patient samples evaluated over three or more experiments for each cytokine and HDACi treatment assessed. Each paired line represents an individual patient sample.

Supplemental Figure 4.

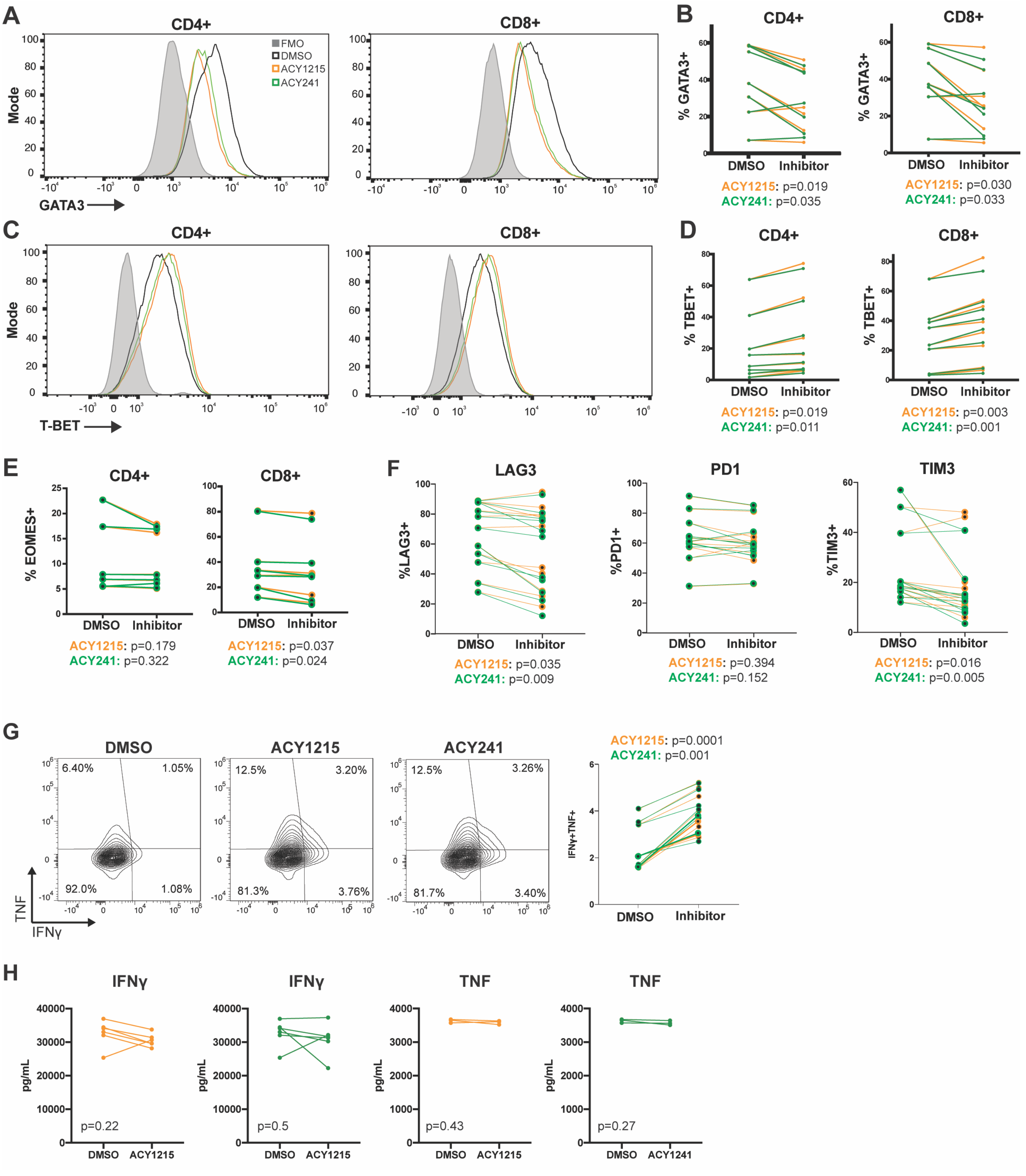

Supplemental Figure 4. ACY-1215 and ACY-241 Effects on Transcription Factor Expression and T-cell Exhaustion. T-cells were expanded with 6,000IU/mL IL-2 for one week in the presence of DMSO, ACY-1215 500nM or ACY-241 500nM. Cultures were activated one day prior to flow analysis. Representative histograms for **(A)** GATA3 and **(C)** T-BET expression in CD4+ and CD8+ T-cells are shown for DMSO (black), ACY-1215 (orange), ACY-241 (green) and fluorescence minus one (FMO) control (grey). CD4+ and CD8+ T-cells expressing **(B)** GATA3 or **(D)** T-BET in DMSO control and paired inhibitor-treated samples are graphed. **(B, D)** Results are shown for at least seven patient samples evaluated over three to four experiments for each marker assessed. Each paired line represents an individual patient sample. **(E)** Paired analysis of EOMES expression is also shown. Results shown are from six patients evaluated in two to three independent experiments for each HDACi treatment. **(F)** Paired analysis of CD8+ T-cells expressing LAG3, PD1 or TIM3 are shown for eleven patients evaluated in three independent experiments. **(G)** Expanded T-cells were activated with αCD3/CD28 Dynabeads for 24 hours. Representative contour plot from one patient and paired analysis of CD8+ T-cells co-expressing IFNγ and TNF are shown for eight samples evaluated in two independent experiments. **(H)** T-cells were expanded with 6,000IU/mL IL-2 for one week in the presence of DMSO, ACY-1215 500nM or ACY-241 500nM. Cells were activated with αCD3/CD28 Dynabeads for additional 72 hours. IFNγ and TNF secretion was assessed by Luminex. Paired analyses are shown for three patient samples assessed for IFNγ production and for six samples evaluated for TNF production across two experiments.

Supplemental Figure 5.

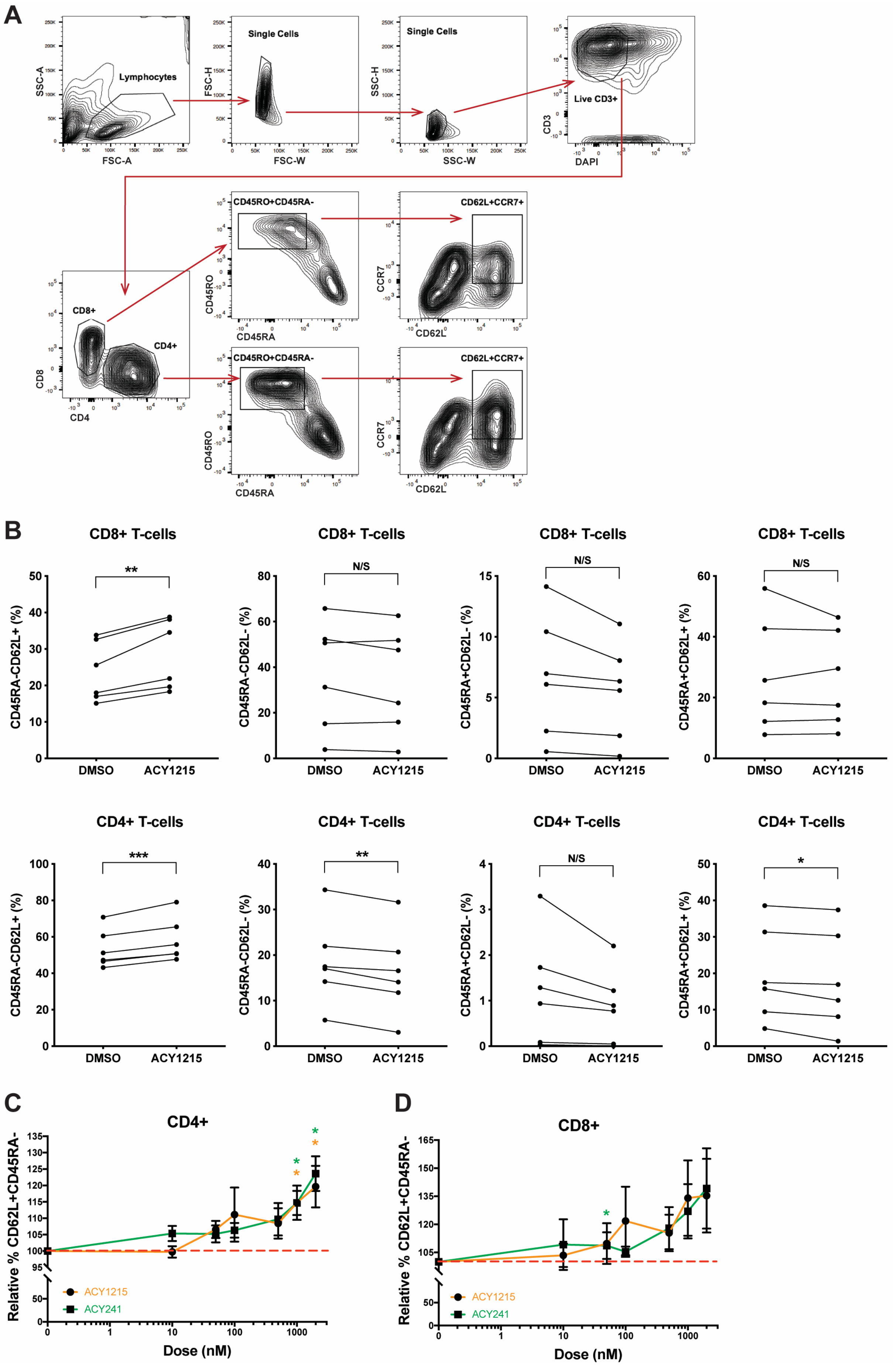

Supplemental Figure 5. Changes in T-cell memory phenotype induced by HDAC inhibition. (A) The general gating strategy for analysis of CD45RO+CD45RA-CD62L+CCR7+ T-cell memory phenotype in flow cytometry experiments is shown. **(B)** T-cells from six melanoma patient PBMC samples were expanded in 6,000IU/mL IL-2 and DMSO or ACY-1215 500nM. Paired analysis for CD45RA-CD62L+ (central memory), CD45RA-CD62L- (effector memory), CD45RA+CD62L (effector)-, CD45RA+CD62L+ (naïve) are shown for CD8+ and CD4+ T-cells from six patients. **(C-D)** Melanoma patient T-cells were cultured in 100IU/mL IL-2 with ACY-1215 (orange lines, circles) or ACY-241 (green lines, squares) for 72 hours. **(C)** CD4+ and **(D)** CD8+ T-cells were assessed by flow cytometry for percentages of CD62L+CD45RA- central memory cells at the indicated concentrations. Mean percent central memory (±SEM) relative to DMSO controls of additional three patient samples assessed is shown on the Y-axis; the dose of inhibitor is indicated on the X-axis. *$p \leq 0.05$, **$p \leq 0.01$, ***$p<0.001$.

Supplemental Figure 6.

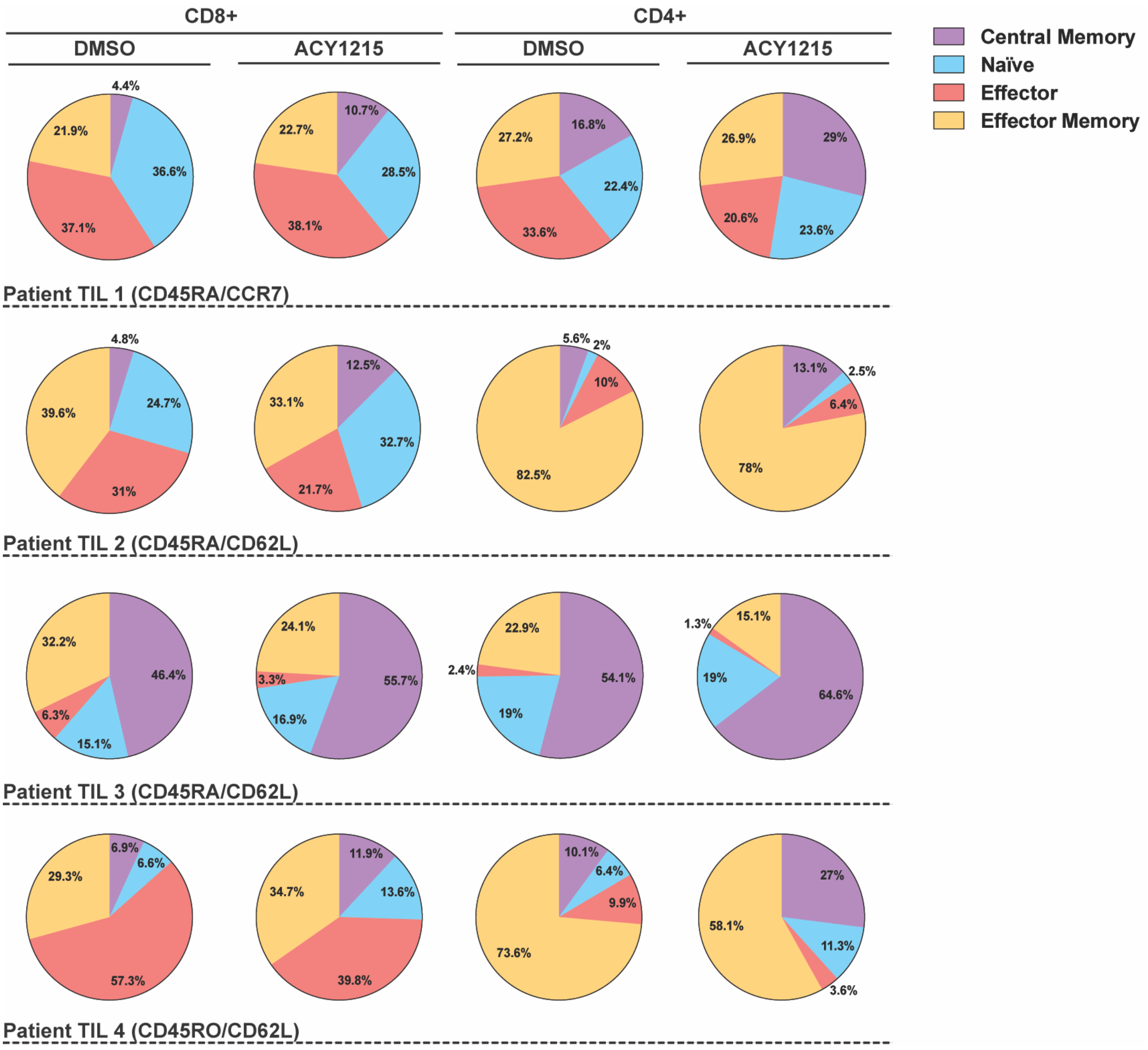

Supplemental Figure 6. Changes in TIL subsets following expansion with HDAC inhibitors. Four melanoma patient-derived TIL samples were expanded across four experiments with 6,000IU/mL IL-2 for one week in the presence of DMSO or ACY-1215 500nM and assessed for CD8+ (left) and CD4+ (right) subsets.

Supplemental Figure 7.

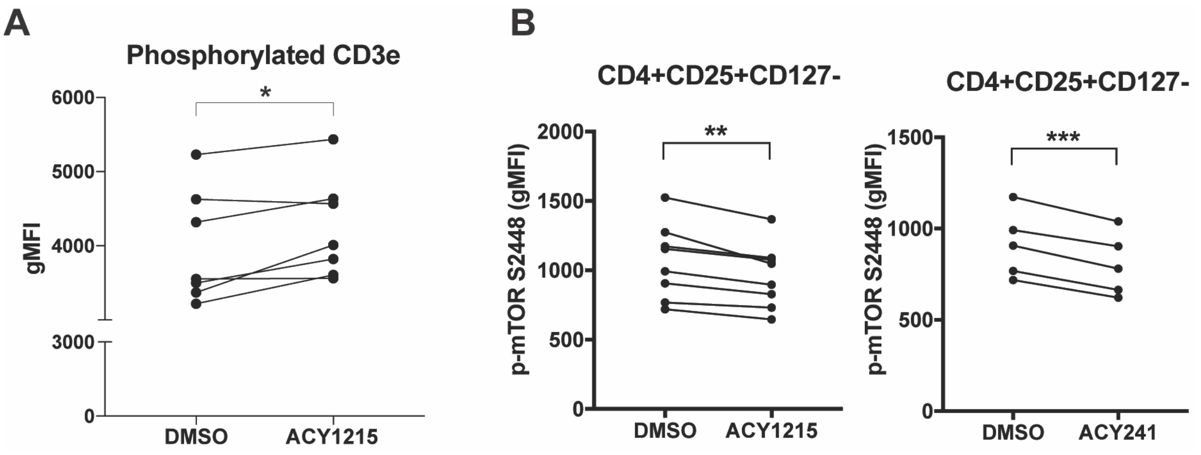

Supplemental Figure 7. ACY-1215 and ACY-241 effects on T-cell signaling. T-cells from melanoma patient PBMC samples were expanded with 6,000IU/mL IL-2 and DMSO, ACY-1215 500nM, or ACY-241 500nM for one week. **(A)** T-cells were activated for 24 hours and evaluated for CD3ε phosphorylation by Luminex. Paired analyses of seven samples assessed over three experiments are shown. **(B)** Non-activated T-cells were evaluated by flow cytometry for phosphorylation of mTOR S2448 in CD4+CD127-CD25+ Tregs. Geometric mean fluorescence (gMFI) is graphed for at least five patient samples assessed in two experiments. *p<0.05, **p<0.01, ***p<0.001.
